# Supplementary material for: Phylogeography of the second plague pandemic revealed through analysis of historical Yersinia pestis genomes
Source: Nat Commun. 2019 Oct 2;10:4470. doi: 10.1038/s41467-019-12154-0 (PMC6775055; doi:10.1038/s41467-019-12154-0)
Supplement: Supplementary file 1 — Supplementary Information [file 41467_2019_12154_MOESM1_ESM.pdf]

Supplementary Information

‘Phylogeography of the second plague pandemic revealed through analysis of historical *Y. pestis* genomes’

Spyrou et al.

Supplementary Note 1

Archaeological context information

"Laishevo III cemetery", Laishevo Republic of Tatarstan, Russia

The Laishevo III cemetery was discovered southeast of the Laishevo town (Laishevo district, Tatarstan Republic, Russia), on the confluence of the Volga and Kama rivers. The site was excavated in 1979, with a large quantity of "Bulgarian" pottery from the Golden Horde period collected from both an eroded part of the site and from burials. It is suggested that the cemetery was located on the site of the settlement, called the Laishevo III settlement. Excavations were carried out along the eroding part of the cemetery. A total of 34 Muslim-type burials were excavated. The individuals were buried in supine position; their heads were oriented to the west, with minor deviations to the south. Hands were usually positioned on the abdomen or the chest. Apart from pottery, few additional artefacts were identified within the burials, such as belt buckles and beads, as well as a single bronze earring. Based on the ceramic findings and the bronze earring, it is suggested that the cemetery most likely dates to the Golden Horde period of Volga Bulgaria (which mainly encompassed the 13th and 14th centuries AD) – though the identified assemblage was suggested to likely not date earlier than the 14th century. Anthropological examination identified skeletal remains of 40 individuals: 14 children of up to 10 years, 13 males and 13 females.

Burial no. 10, where individual LAI010 was discovered, was partially destroyed, and had a depth of 40 cm. Two skeletons were found in this burial. Individual LAI010 (skeleton A from burial 10) was located in the southern part of the grave pit, and only the upper part of the skeleton was preserved. Through anthropological examination, the individual was suggested to be a 35-45 year-old male.

The triple burial 27, where individual LAI009 was found, was also at a depth of 40 cm. The skeletal remains of individual LAI009 (skeleton B from burial 27) were not well preserved. The skeleton was disarticulated with the skull found on the western part of grave pit and the remaining skeletal elements found between skeletons A and C. An iron buckle was found inside the skull, and the only bronze earring discovered on this site was also found at closest proximity to this skull than any other skeleton. Although from anthropological examination the individual was suggested to be a male of 30-40 years of age, genetic sexing suggests that it belonged to a female.

"16 rue des Trente Six Ponts", Toulouse, France,

The archaeological rescue excavation that took place at 16 rue des Trente Six Ponts in Toulouse has uncovered a funerary space used between the 5th century and the end of the 14th century. The

funeral area of the Late Middle Ages is the most important as it consists of 109 graves that include 29 multiple graves for a total of 444 individuals of which 306 are buried in three mass graves.

The medieval funeral complex corresponds to the eastern extension of Saint-Michel graveyard discovered in 2002[29]. This cemetery is divided into two parallel rows. These rows are separated by a vacant space, without graves or archaeological structures, allowing movement within the funerary area. The three mass graves which are characteristic of so-called "mortality crisis" graves, are found at the eastern ends of these rows. The graves can be dated by several methods. First, the orientation of the burials corresponds to the one observed for the Late Middle Ages at the excavation of the Saint-Michel cemetery in 2002[29]. Second, the artefacts uncovered in the deposits within the same strata as these graves can be dated between the second half of the 13th century and the end of the 14th century.

Moreover, some dating evidence allows us to understand more precisely the evolution of this funeral complex in the Middle Ages. A first period, the end of the 13th century to the first half of the 14th century corresponds to the beginning of the use of this space as a cemetery. This first phase is dated by a few coin hoards excavated with the buried bodies. Two graves contain coins from the end of the 13th century (1245-1270). Six burials were dated by ^{14}C, three of which belong to a period between the second half of the 13th century and the first quarter of the 14th century.

The second phase corresponds to a "crisis" cemetery, probably linked to a plague epidemic that affected Toulouse between 1347-1350. This hypothesis is supported by several coin finds. Their contemporaneity with the Black Death can be demonstrated by depositions within a double burial (SP2806) as well as in two of the mass graves. The first deposit is found within the double burial and consists of 34 “double tournois” of Philippe VI and of two “double parisis” of Philip VI that were found to be associated with one of the bodies. These coins are thought to have been issued between March 1347 and August 1348. The second deposit, related to the second burial, consists of two “double parisis” of Philip VI issued between April 1347 and August 1350. The third deposit is associated with one of the mass graves (SP1827) and contains 36 coins (“double tournois” and “double parisis” of Philippe VI) also issued between March 1347 and August 1348. Finally, the second mass grave (SP3408) contains a deposit of four “double tournois” of Philip VI issued between August and December 1348.

The grave SP1350 corresponds to a double burial dug in stratigraphic deposits post-dating the 13th century and has the same orientation as the other multiple graves dated to the same period. It is therefore contemporaneous with the plague episode that affected Toulouse during the late 1340s. The first, skeleton SQ1352 (TRP002), corresponds to an adult woman, aged 20 to 49 years, buried at the same time as the individual SQ1353, identified as an infant (0-1 years). Skeleton SQ1352 was

buried in a flexible shroud type material unlike SQ1353, which does not appear to have been similarly treated. Both individuals were likely buried in a coffin.

"The New Churchyard", London, England

Population growth in 16th-century London, coupled with a severe outbreak of plague in 1563, led to the 'New Churchyard' opening in 1569, a municipal, non-parochial burial ground[30]. The chosen site was outside the north wall of the city, on land, which had previously been part of the priory of St Mary Bethlehem, later better known as 'Bethlem' or 'Bedlam' Hospital. Indeed, the ground was often referred to as the 'Bethlem' or 'Bedlam' burial ground because of its location.

When the New Churchyard closed to burials in 1739, it was densely packed with graves primarily including the remains of the city's poor and those on the fringes of society. The use of the ground covered a period in which London suffered several plague epidemics, particularly in 1603, 1625, 1636 and 1665, with parish records confirming that hundreds of victims of the disease were sent to the New Churchyard for burial.

During the Crossrail Central development at the Broadgate ticket hall worksite at Liverpool Street, London, in 2011–15, archaeological investigations by MOLA (Museum of London Archaeology)[31] resulted in the excavation of 3354 of the estimated *c.* 25,000 burials within the New Churchyard. This work provided an opportunity to explore health and disease of the city's inhabitants during a period of considerable population growth fed by migration to the expanding metropolis.

The discovery of a mass pit in the central area of the southern half of the burial ground, containing at least 42 individuals, provided an opportunity to investigate the first archaeologically excavated 17th-century plague burial in London. The pit contained stacks of coffined and un-coffined burials, up to eight individuals deep. It was filled in a single event. The head ends of the coffins were alternated to allow the maximum use of space within the pit. A single perpendicular line of burials filled a gap at one end.

The archaeological dating, although imprecise and complicated by some intrusive finds introduced by later grave cuts, is consistent with a late 16th- or early 17th-century date. The east-west alignment of the pit was typical of burials from the earlier period of the burial ground's use, 1569 – 1670. The fill contained a small pottery assemblage dated between 1550 and 1610, and coffins within the pit were of a type that appeared in the last quarter of the 16th century and was ubiquitous from 1650 onwards. While radiocarbon dating was not sufficiently precise to distinguish between specific plague events, the dates clearly indicated that the Great Plague of 1665 was too late to have been responsible for the mass burial and the outbreaks in 1603, 1625 and 1636 were the most likely.

Augustinian Friary, Cambridge, England

Documentary evidence indicates that the Augustinian Friary in Cambridge was founded between 1277 and 1289, as a mendicant institution with a strong focus on learning and continued until the Dissolution of the Monasteries in 1538. The friary probably acquired burial rights in 1290 if not earlier and in 1302 these were extended to include individuals who were not members of the Augustinian order. Excavations in 2016–17 by the Cambridge Archaeological Unit as part of the redevelopment of the New Museums site by the University of Cambridge revealed two phases of friary related activity, both with associated burials[32].

The earlier phase mainly comprised a cemetery located south of the friary church, where 32 burials that appear to be a mixture of friars and lay individuals were excavated. General archaeological evidence, including belt buckles associated with some of the burials, suggests that the cemetery spans the late 13th to mid-14th century, with radiocarbon dating of three burials in a stratigraphic sequence indicating that burial continued until at least the 1330s (all radiocarbon dates quoted to 95% probability). Ancient DNA analysis of one of the skeletons from the cemetery F.355 (NMS003) yielded a whole *Y. pestis* genome that likely has an identical genotype to other European genomes attributed to the Black Death episode (see Supplementary data 3), indicating that this burial likely dates to 1348/49. Stratigraphically F.355 (NMS003) predates two radiocarbon-dated burials F.332 and F.328. F.332 dates to 1286–1397 cal AD, but when factors including the marine reservoir effect, bone turnover/remodeling and stratigraphic sequence are considered it is dated to 1323–1426.

The second phase related to the friary is marked by the construction of the claustral ranges. Typologically the cloister arcade is dated to between *c*. 1330 and *c*. 1390. This construction is unlikely to have predated the late 1330s, as it was not until then that the friary managed to acquire enough properties to embark on this major project. Additionally, the legs of F.355 (NMS003), which was likely buried in 1348/49, were removed when the foundation trench was dug for the wall of the cloisters. As this process did not disturb the rest of the bones it is probable that some time had passed since the interment, conservatively suggesting a date of no earlier than *c*. 1360 for the wall footing.

Six individuals were buried in the Chapter House in the eastern claustral range of the friary *c*. 1360/90–1538. As with the earlier cemetery the burials appear to be a mixture of friars and lay individuals. Three of the burials from the Chapter House tested positive for *Yersinia pestis*; F.230 (NMS002) yielded a whole-genome, whereas F.190 (NMS001) and F.310 (NMS005) remain to be confirmed. There is no stratigraphic evidence to indicate the relative sequence of these three burials and the *Y. pestis* DNA could relate to either a single plague outbreak or multiple outbreaks. F.230

(NMS002) has a copper alloy symmetrical double oval frame belt buckle, dating to between *c*. 1350 and the 16th century and the grave fill contained some 15th- to 16th-century lead window came. F.230 has been radiocarbon dated to 1437–1619 cal AD, but when various factors are allowed−including the marine reservoir effect, the bone turnover/remodeling, the stratigraphic sequence and the fact that the burials date to 1538 at the latest−the probable date range is 1475–1536. There are several documented plague outbreaks in England during the late 15th and early 16th centuries, with major outbreaks in 1471 and 1479–80 and smaller scale recurrences in 1499–1500, 1509–21, 1526–32 and 1535–36, though it is uncertain which of these outbreaks affected Cambridge. The only specific dating evidence associated with F.190 (NMS001) is a small amount of pottery that indicates a 16th century date, and the location of the grave suggests that it may be contemporary with F.230 (NMS002). F.310 is also accompanied by a copper alloy symmetrical double oval frame belt buckle, dating to between 1350 and the 16th century.

"Sankt Johans Freidhof", Nabburg, Germany,

The "Sankt Johans Freidhof" churchyard (sic, see Hensch 2014)[33] in the city of Nabburg in Southern Germany was excavated between 2008 and 2012 next to the hospital church "St. Maria", consecrated in 1420. However, the earliest graves are attributed to the older parish church "St. Johannes der Täufer" from the end of the 13th century. The ~200 excavated graves were found in a maximum of eight layers and could be classified in two groups: The older graves from the late 13th to late 14th centuries are dug in a strict and regular layout, the younger graves from the 15th century to the closure in 1597 are more irregular, presumably due to the lack of space. Only in four cases, the use of a coffin could be attested by the finds of iron nails and wooden remains.

Towards the western border of the cemetery, in total nine multiple burials were found with between two and four individuals. The four individuals that tested positive for *Y. pestis* originate from three multiple burials: NAB002 (arch. ID 451, early juvenile male) and NAB003 (arch. ID 452, early adult female) were found in a triple burial with an additional young woman, all piled up in a narrow grave pit in supine position. NAB004 (arch. ID 457, presumably female of 6-12 years, supine position) was buried in a simultaneous double burial together with an adult female on top in the opposite orientation. NAB005 (arch. ID 471, early mature female) was also buried in a simultaneous double burial, here with an adult male individual beneath in prone position.

A connection to the second plague pandemic was suggested by the archaeologists given the grave characteristics, despite the fact that there are no historical records for the Black Death in Nabburg. However, epidemic records exist for the nearby towns of Amberg, Sulzbach, Burglengenfeld and Regenstauf from 1349 onwards.

St. Leonhardi, Manching-Pichl, Germany

During the renovation works of 1984/85 at St. Leonhardi, a mass grave was found under the sacristy. The mass grave revealed a minimum number of 75 individuals in four layers. The construction of the mass grave suggests that it was not dug into the ground but the individuals were placed on ground level without coffins and afterwards covered with earth[28]. However, the removal of the ground floor within the nave also revealed the remains of six additional individuals with some of them considered to be the church donors. The high amount on disarticulated skeletal elements within the mass grave hints towards a more intensive use of the site as a burial ground. The construction of the sacristy can be dated to the second half of the 15th century, which would give a *terminus ante quem*, assuming that the sacristy was built after the deposition. Radiocarbon dates of earlier studies gave two contradicting intervals, one spanning roughly the 12th century and another spanning the 14th century[25-27], which could be explained by erroneously assigned scattered remains of earlier burials in this area.

The mass grave was repeatedly subject to aDNA studies that investigated the presence of *Y. pestis* DNA with PCR and qPCR assays[25-28,34]. In this study, individuals MAN008 (arch. ID MPS03-IV, adult female) and MAN015 (arch. ID MP56-X) tested positive for *Y. pestis* through qPCR and high-throughput sequencing (Supplementary table 2). Due to the apparently difficult assignment of skeletal elements to the individuals in the mass grave, only teeth in situ were sampled, so the jawbone could be used for ^{14}C dating (Supplementary table 1). The resultant ^{14}C dates (2-sigma: 1283-1390 calAD), revealed a 14th century interval for the deposition. Plague is thought to have first entered Bavaria in 1348 during the Black Death and continued to affect this region with epidemics during the second half of the 14th century, after the Black Death. Based on our current genomic data, the MAN008 isolate from Manching is genetically divergent from all other Black Death genomes from Europe, including one from the nearby city of Nabburg (Figure 2). As such, these results are in support of MAN008 being representative of a post-Black Death outbreak that occurred in Bavaria likely during the second half of the 14th century.

Possenhofener Str. 3, Starnberg, Germany,

From 2007 to 2009 the southern and southeastern parts of the churchyard of the former parish church St. Benedikt in Achheim – todays Starnberg (Upper Bavaria) – were excavated. St. Benedikt was first mentioned in 1226 and demolished between 1764 and 1816[35]. The excavation revealed 365 burials, estimated to represent only one third of the cemetery and dating mostly between the 8th/9th and the 18th centuries. However, five stone plate graves date between the middle of the 7th and the early 9th centuries. This indicates, that the cemetery was originally founded as a private

burial ground by an aristocratic family, probably living nearby in a manor, in the mid-7th century. The first church can be dated to the middle of the 7th century, too, and is actually one of the oldest known churches in Bavaria.

After the church was donated to Benediktbeuren Abbey in the 8th or 9th century, it was turned into a parish church for the surrounding village of Achheim. The Merovingian church was replaced by a larger one in the late 10th or early 11th century. This medieval church was once again completely rebuilt in the first half of the 15th century.

Achheim stayed a small village at the shore of Lake Starnberg until the mid-19th century, when it became part of the growing modern town of Starnberg. Starnberg Castle (first mentioned in 1245), which surmounts the former village of Achheim, was a temporary summer residence of the dukes of Bavaria during late medieval and early modern times, especially in the 15th and 16th centuries. During this period, several members of the aristocracy, closely connected to the ducal court or the administration of the castle, were buried in a side chapel of St. Benedikt. A couple of parish priests were buried in the church, too. Nevertheless, most burials can be identified with the common population of Achheim and probably some outlying homesteads – mostly fishermen, peasants or craftsmen. Thus, St. Benedikt represents a typical parochial churchyard of a rural community in Southern Bavaria.

The medieval graves are characterized by the complete absence of grave goods and can only be dated by stratigraphy, radiocarbon analysis and their arm posture. In the years around 1300 burial rites changed and few grave goods can be found again, especially rosaries as well as occasional coins, buckles or finger rings.

One triple burial – situated just a few meters southeast of the sacristy and belonging to the late medieval period – was the only multiple burial found at this site. Due to this, it was examined here for the presence of *Y. pestis*. Individual STA001 (arch. ID 207, infans II to juvenile, aged 12–14, presumably male) tested positive for the bacterium through qPCR and high throughput sequencing (Supplementary table 2). In its hands the individual held a rosary, as shown by few remains of blue glass beads, which cannot be dated precisely. The juvenile was simultaneously buried together with two other children aged 3–5 (arch. ID 208, infans I) and 12 (arch. ID 214, infans II), in regular supine position. The rosary found with STA003 (arch. ID 214) can be dated between the late 14th and the early 16th centuries. This special long form of a rosary was composed of approximately 150 ring-shaped bone beads (15 mm in diameter), represented by 122 intact beads and 38 fragments. Taking into account the 1-sigma interval of the radiocarbon dating, the date of the triple burial can be narrowed down to 1433–1494 (59,6 %), respectively to 1420–1523 (74,5 %) when considering

the 2-sigma interval. Thus, these three subadults died during a historically undocumented plague outbreak in the 15th or early 16th century.

Kirchhof St. Johannis, Landsberg am Lech, Germany

After Landsberg was granted the town privilege in 1315 the small trading post on the crossroads of the salt route soon started to prosper[36]. Despite the outbreak of the plague in 1349 the town's population grew and at the beginning of the 17th century it reached an estimated number of around 4500 inhabitants. To take care of the salvation of the growing population the parish church was extended in the mid-15th century, resulting in a shortage of available burial space. As a result, a new cemetery was established within the town walls. The usage time of the St. Johannis churchyard could be narrowed down by historical documents to between 1507 and 1806. In this timespan Landsberg witnessed several plague epidemics: 1586, 1592, 1607, 1627/28 and 1634. While there are no mortality estimates available for the 16th century epidemics, in 1607 74 victims were counted. From September 1627 until January 1628 plague claimed 284 victims and in 1634 760 deceased (including a large number of non-locals) were registered at the town's obituary columns. It is estimates that epidemics as well as the Thirty Years' War reduced the population by almost 80%.

Partially excavated between 2015 and 2016, the churchyard of St. Johannis revealed remains of more than 900 individuals[37,38]. On the lowest of eight burial levels, six multiple burials with four to eleven individuals were found. All together the mass graves revealed 47 individuals in up to four layers. Within the multiple burials, an equal proportion in the number of males and females can be observed; specifically, six individuals could be determined as male, eight as likely male, ten as female and further seven as likely female. 16 individuals are of indeterminate morphological sex (mostly subadults). Furthermore, in most of the multiple burials and one single burial (likely female, 11 to 14 years) the individuals were laying in or were covered with quicklime. In combination with the high percentage of subadult individuals (nearly 60%) and the absence of any evidence for trauma on the skeletons, an epidemic context was suspected by the excavators.

Imprints in the quicklime show that the dead were wrapped in shrouds and were then piled up in the burial pits. In contrast to the regular burials, which contained the typical furnishing (rosaries and multiple types of devotional objects) of catholic funerals of that period, just a few non-datable finds came from these epidemic burials.

Three individuals tested qPCR positive for *Y. pestis*. These were LBG002 (arch. ID 460, presumably male juvenile) was buried in a 11-fold burial, LBG005 (arch. ID 572, presumably

female 6-12 years) from a single burial with quicklime, and LBG007 (arch. ID 598, presumably a female juvenile) buried in a quintuple burial with quicklime.

Nägeligasse, Stans, Switzerland

The partial excavation of the churchyard of "St. Peter und Paul" took place between 2015 and 2016. The cemetery was in use from the 8th century until the 1850s AD. Besides 122 single burials, the excavation revealed three multiple burials with a minimum number of four (pos. 369 – multiple burial 3), 16 (pos. 230 – multiple burial 1) and 26 individuals (pos. 232 – multiple burial 2) respectively[39,40]. The multiple burial pits were located at the margin of the cemetery, with individuals being buried in supine extended position mainly in a west-east orientation. The excavators were able to reconstruct that the individuals were likely buried simultaneously within a large pit, including some burial planks. Metal hooks indicate that the individual were buried in clothing. The excavation was accompanied by physical anthropologists that sampled the individuals on site (1 tooth per individual). A publication about the multiple burials anthropological analysis, including radiocarbon data is currently in preparation (Somers et al., in prep.)

Historical sources report the presence of plague during a minimum of seven succeeding outbreaks between 1493 and 1630 in Stans[39]. However, none of the recorded outbreaks could be assigned specifically to the multiple burials. The present molecular results suggest that individuals from all three multiple burials were positive for the *Y. pestis* bacterium. These were: STN002 (arch. ID 71), STN004 (arch. ID 91), STN005 (arch. ID 92), STN007 (arch. ID 97/251), STN008 (arch. ID 98/250), STN011 (arch. ID 102) and STN012 (arch. ID 103) from multiple burial 1; STN013 (arch. ID 85), STN014 (arch. ID 104), STN015 (arch. ID 105), STN016 (arch. ID 106), STN018 (arch. ID 121), STN019 (arch. ID 123), STN020 (arch. ID 124), STN021 (arch. ID 125) and STN026 (arch. ID 134) from multiple burial 2; as well as STN031 (arch. ID 165) and STN032 (arch. ID 167) from multiple burial 3.

Domlinden 12*, *Brandenburg an der Havel, Germany

During a survey in 2011, the burial of three individuals was found on the "Dominsel", the centre of the old town of Brandenburg, situated between two streams of the Havel. It is noteworthy that no context of a contemporary cemetery was found[41]. Instead, it appears that the individuals were buried in the backyard of a bourgeois house. One of the individuals was buried with a clay pipe bowl with the initials of the Dutch manufacturer Samuel Collier, setting the *terminus post quem* between 1630 and 1640. All three individuals were morphologically identified as adult males. Moreover, one of them showed a healed impression fracture of the skull, indicative of interpersonal

violence. Due to the poor health status and the hasty burial that did not follow the Christian burial practices, the individuals have been characterized as foreigners with low social status. Indeed, the oxygen and strontium isotope data show a non-local signature consistent with Scandinavia or the Baltic region. Therefore, it was hypothesized that the individuals were foreign soldiers housed with civilians during the occupation of the city by Swedish troops between 1626 and 1631 as part of the Thirty Years' War (1618 - 1648 AD). Plague waves have been reported for Brandenburg a. d. H. in 1625-1627 and 1631, supporting the year 1631 for the burial.

Y. pestis DNA was identified before in these individuals based on PCR SNP typing[26,27]. Here, we reconstructed whole genomes from two of those individuals, namely BRA001 (arch. ID 1, late adult male) and BRA003 (arch. ID 3, 18-20 years old male).

Supplementary Figures & Tables

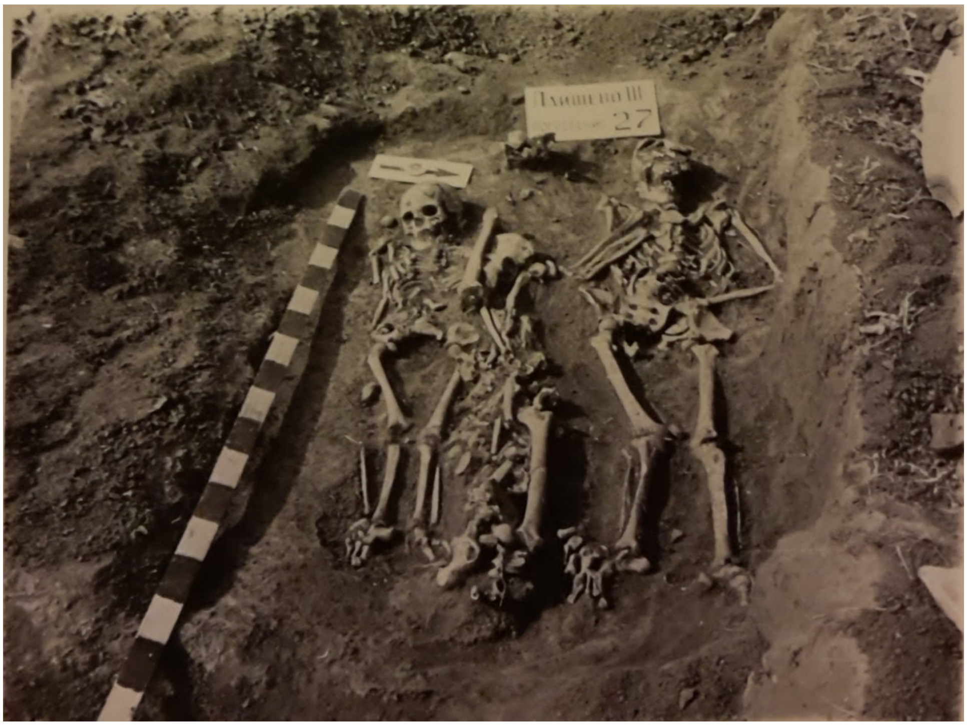

Supplementary figure 1 – The picture shows the triple burial no. 27, where individual LAI009 was unearthed, in the Laishevo III cemetery, Laishevo, Russia. Picture credits: Rafail' M. Fattahov

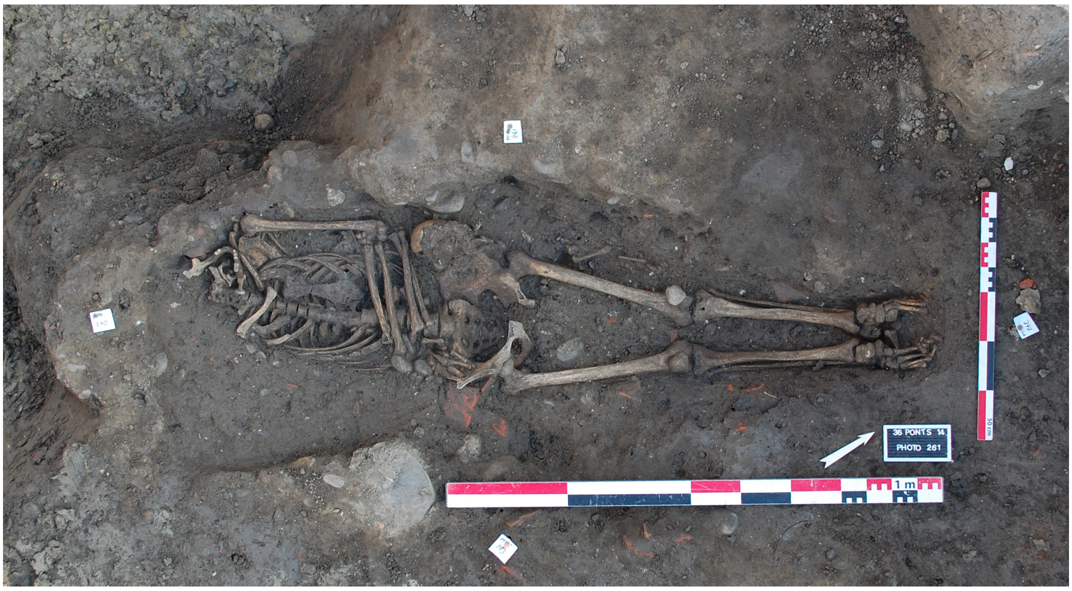

Supplementary figure 2 – Picture of double burial from the Toulouse archaeological site "16 rue des Trente Six Ponts" showing individuals SQ1352 (TRP002) and SQ1353. Picture credits: Archeodunum SAS, Gourvennec Michaël.

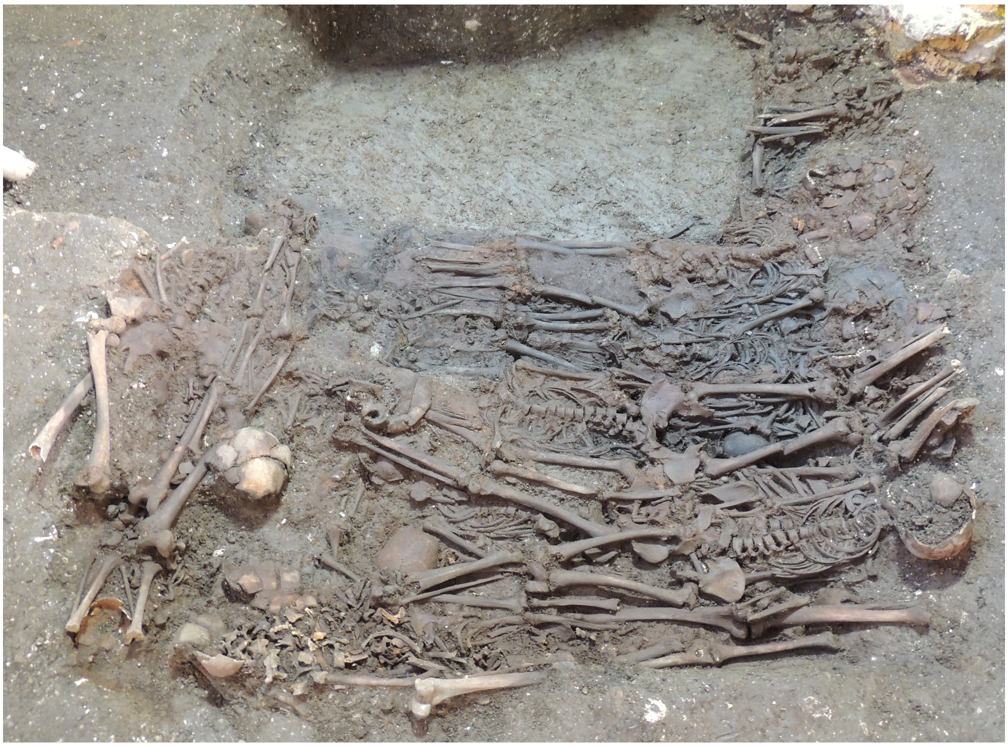

Supplementary figure 3 – The picture shows the mass grave identified in the New Churchyard cemetery in London, England, including all the BED individuals described in this study. Picture credits: Crossrail / courtesy of Museum of London Archaeology (MOLA).

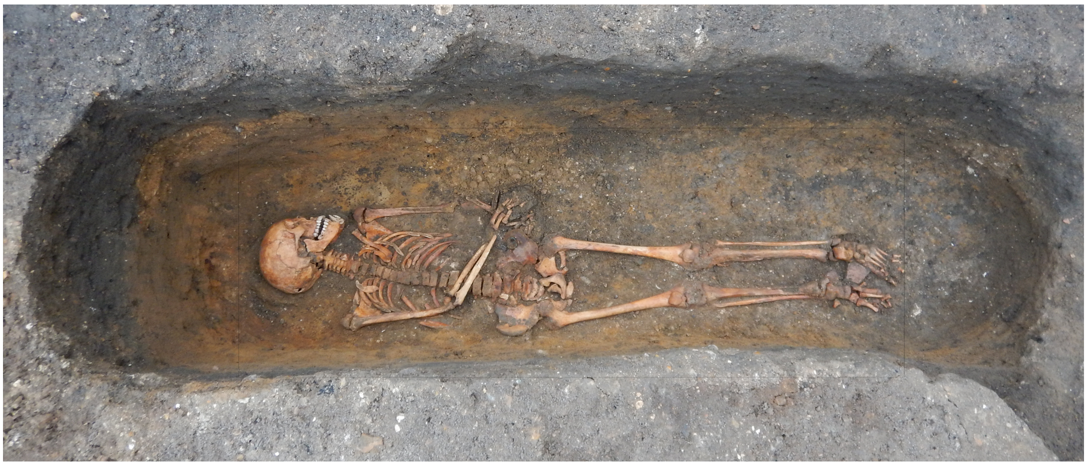

Supplementary figure 4 - The burial of individual F.230 (NMS002) unearthed at the Augustinian Friary, as part of the New Museums site in Cambridge, England. Picture credits: Cambridge Archaeological Unit

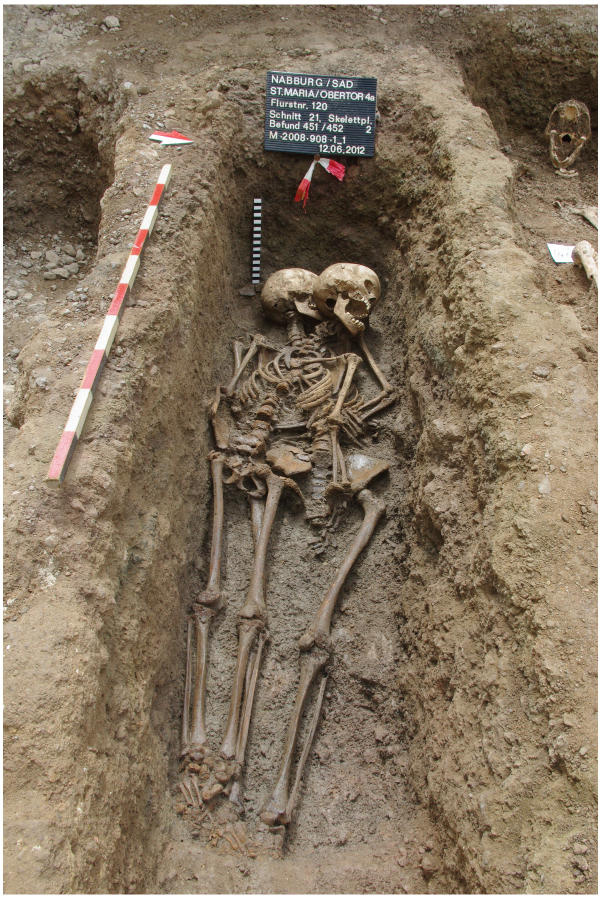

Supplementary figure 5 - The picture indicates the double burial with individuals 451 (NAB002) and 452 (NAB003) unearthed at the Sankt Johans Freidhof in Nabburg, Germany. Picture credits: Mathias Hensch (Schauhütte archaeology)

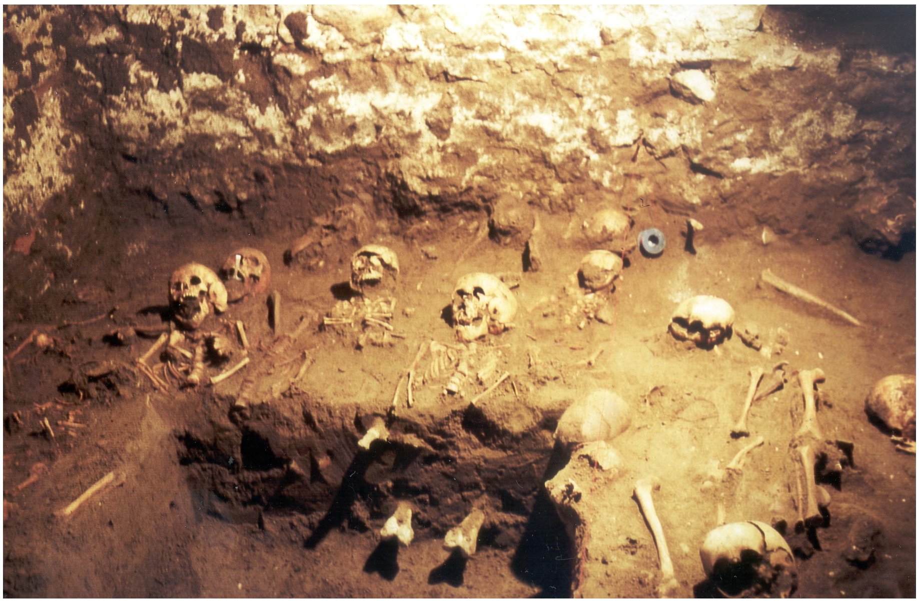

Supplementary figure 6 – The picture indicates the mass burial discovered at St. Leonhardi, Manching-Pichl, Germany, including individuals MAN008 and MAN015. Picture credits: Bavarian State Department of Monuments and Sites

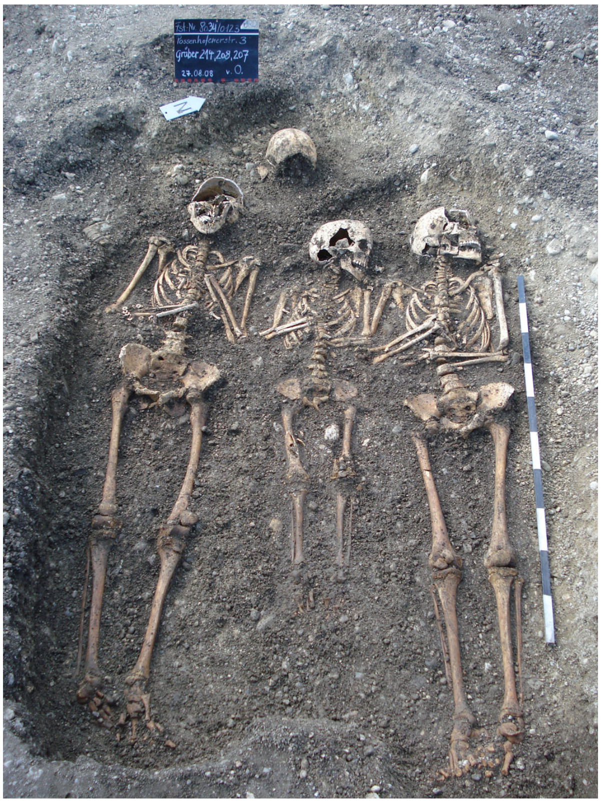

Supplementary figure 7 – Picture of the triple burial (including individual STA001) discovered next to the former parish church of St. Benedikt, Starnberg, Germany. Picture credits: Bavarian State Department of Monuments and Sites

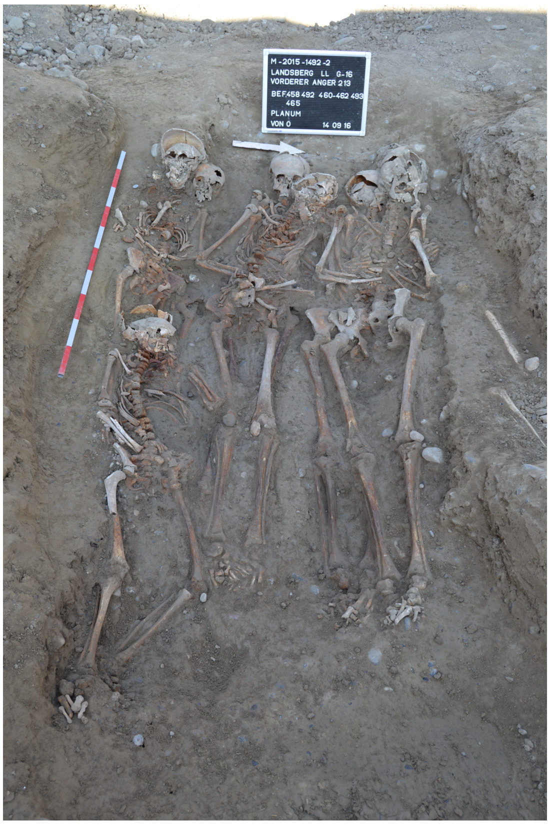

Supplementary figure 8 – Picture of a multiple burial (including individual LBG002) discovered at the St. Johannis churchyard in Landsberg am Lech, Germany. Picture credits: J. Schreiber (Dig it!)

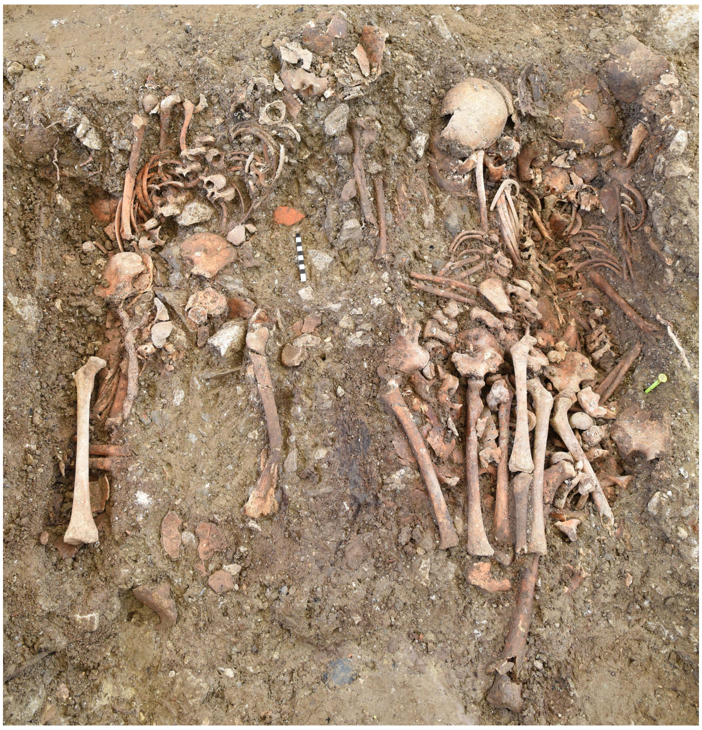

Supplementary figure 9 - Picture of multiple burial 1 (Pos. Nr. 230) unearthed in Nägeligasse, Stans NW, Switzerland, which included individuals STN002, STN004, STN005, STN007, STN008, STN011 and STN012. Picture credits: Department of Physical Anthropology, Institute for Forensic Medicine, University of Bern

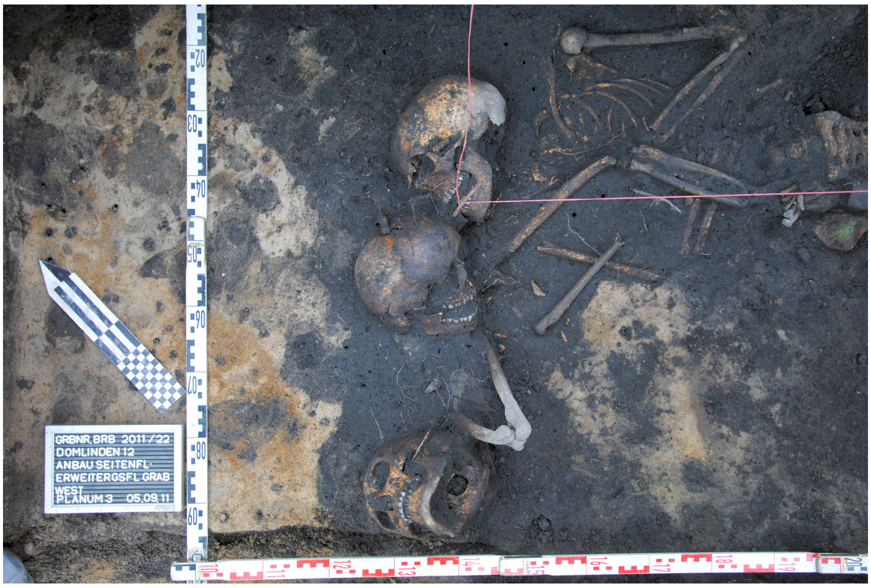

Supplementary figure 10 – Picture of triple burial (including individuals BRA001 and BRA003) discovered in Brandenburg an der Havel, Germany, which has been attributed to the period of the Thirty Years War (1618-1648 AD). Picture credits: S. Dalitz (Untere Denkmalschutzbehörde der Stadt Brandenburg an der Havel).

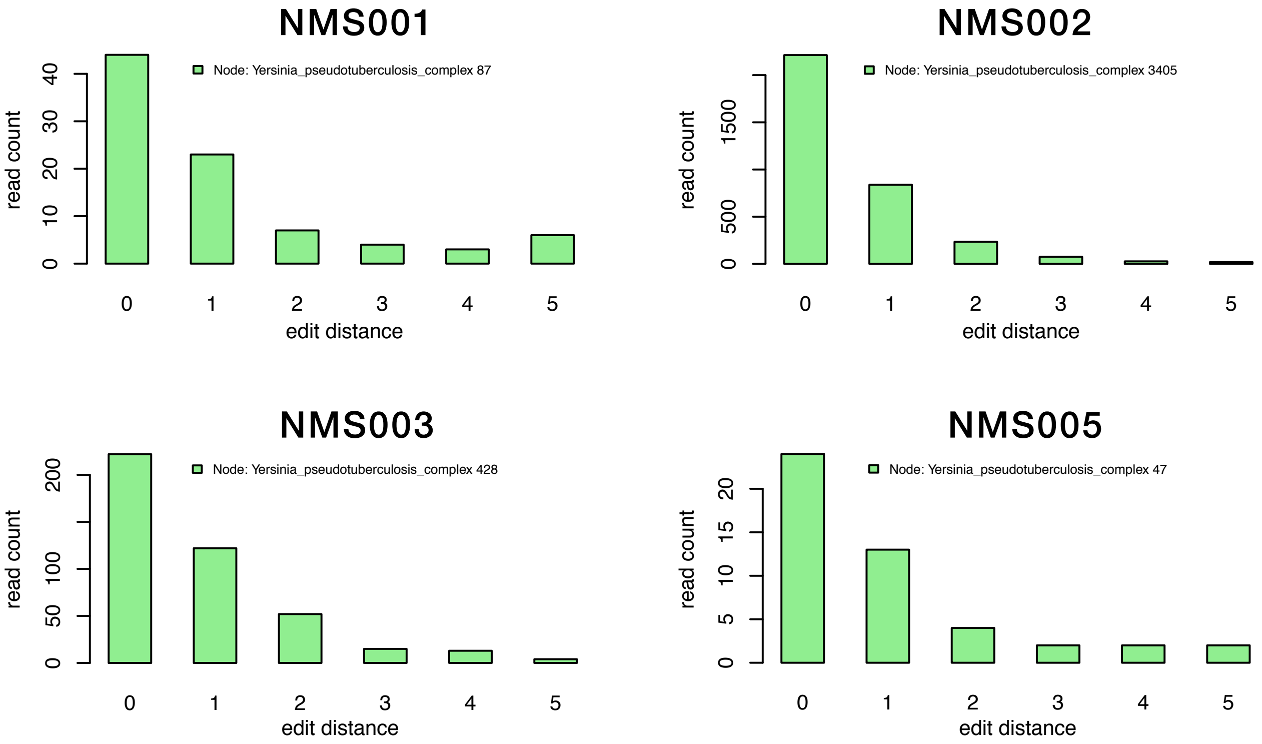

Supplementary figure 11 – Edit distance histograms of shotgun NGS reads calculated with HOPS[1] for all NMS (Augustinian friary) specimens that yielded putative *Y. pestis* matches. The edit distance is calculated for each dataset according to the closest matching reference genome within the *Y. pseudotuberculosis* complex.

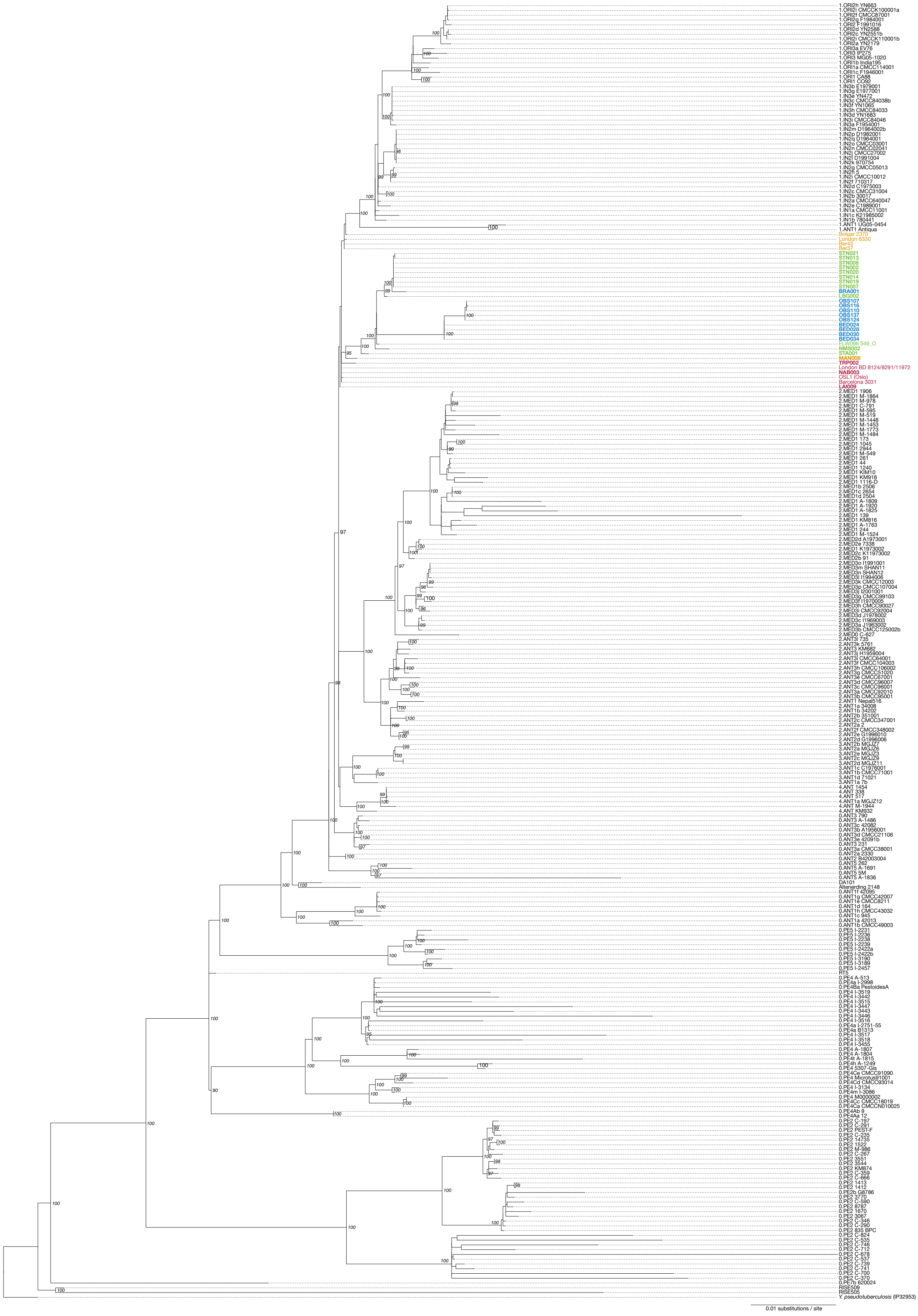

Supplementary figure 12 - Maximum likelihood phylogeny[2] (97% partial deletion) of all *Y. pestis* genomes used in this study. A total of 6,058 SNP positions were considered for the phylogeny. The tree is comprised of 233 modern isolates[3-9], 35 second pandemic isolates, one first pandemic isolate[10], one 2nd- to 3rd-century isolate[11], three Bronze Age isolates[12,13], and a *Y. pseudotuberculosis* isolate (IP32953)[14] that was used as outgroup for rooting the tree. Bootstrap values of 95 or higher are shown. All early 14th-century genomes are shown in red, late 14th-century genomes are shown in orange, 15th-17th century genomes are shown in green and 17th- to18th-century genomes are shown in blue.

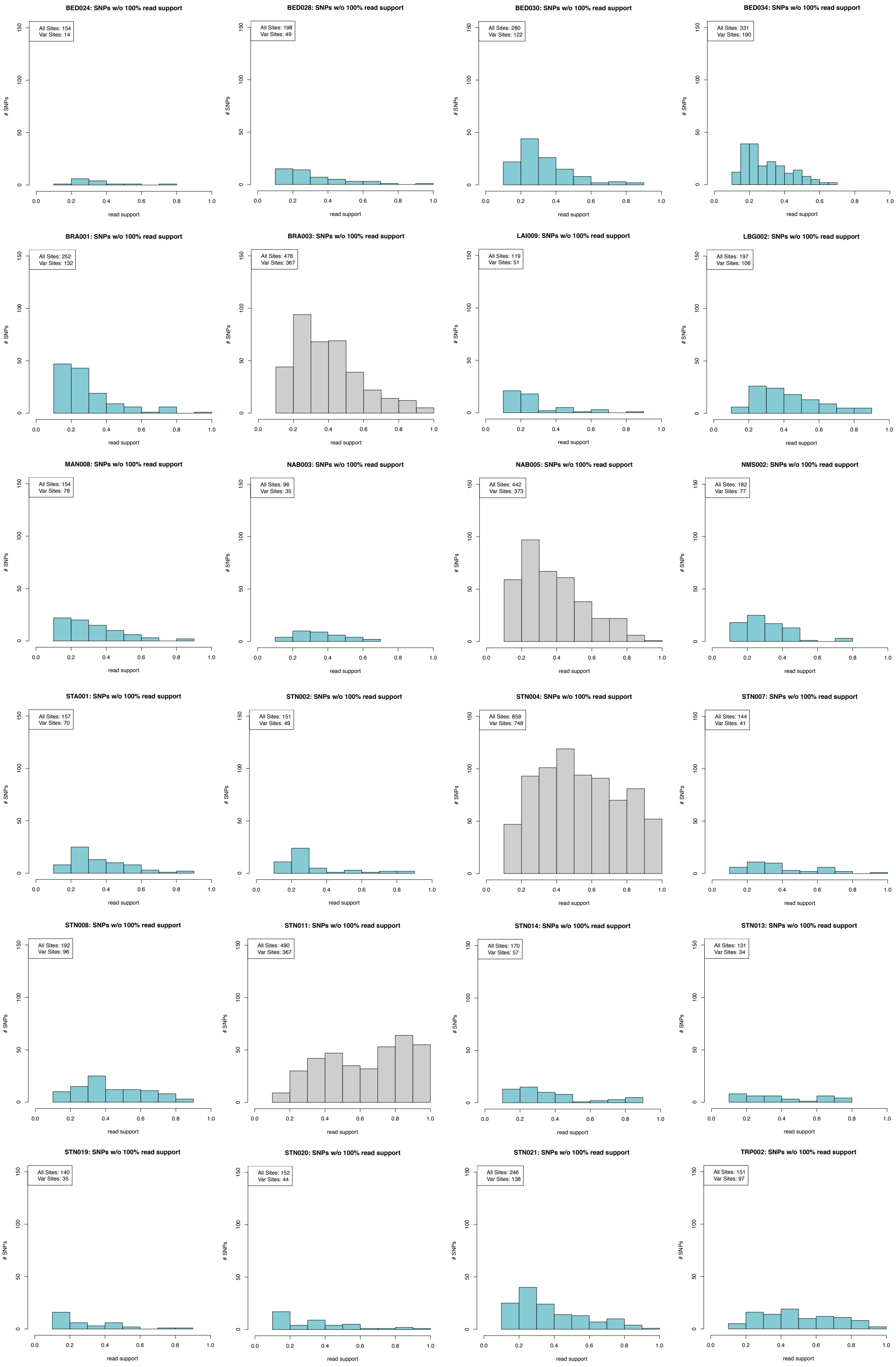

Supplementary figure 13 – Histograms showing the distribution of SNP allele frequencies in newly produced *Y. pestis* genomes with ≥5-fold coverage. The histograms were constructed using R version 3.4.1[15]. Histograms shown in grey represent genomes that were excluded from subsequent analyses.

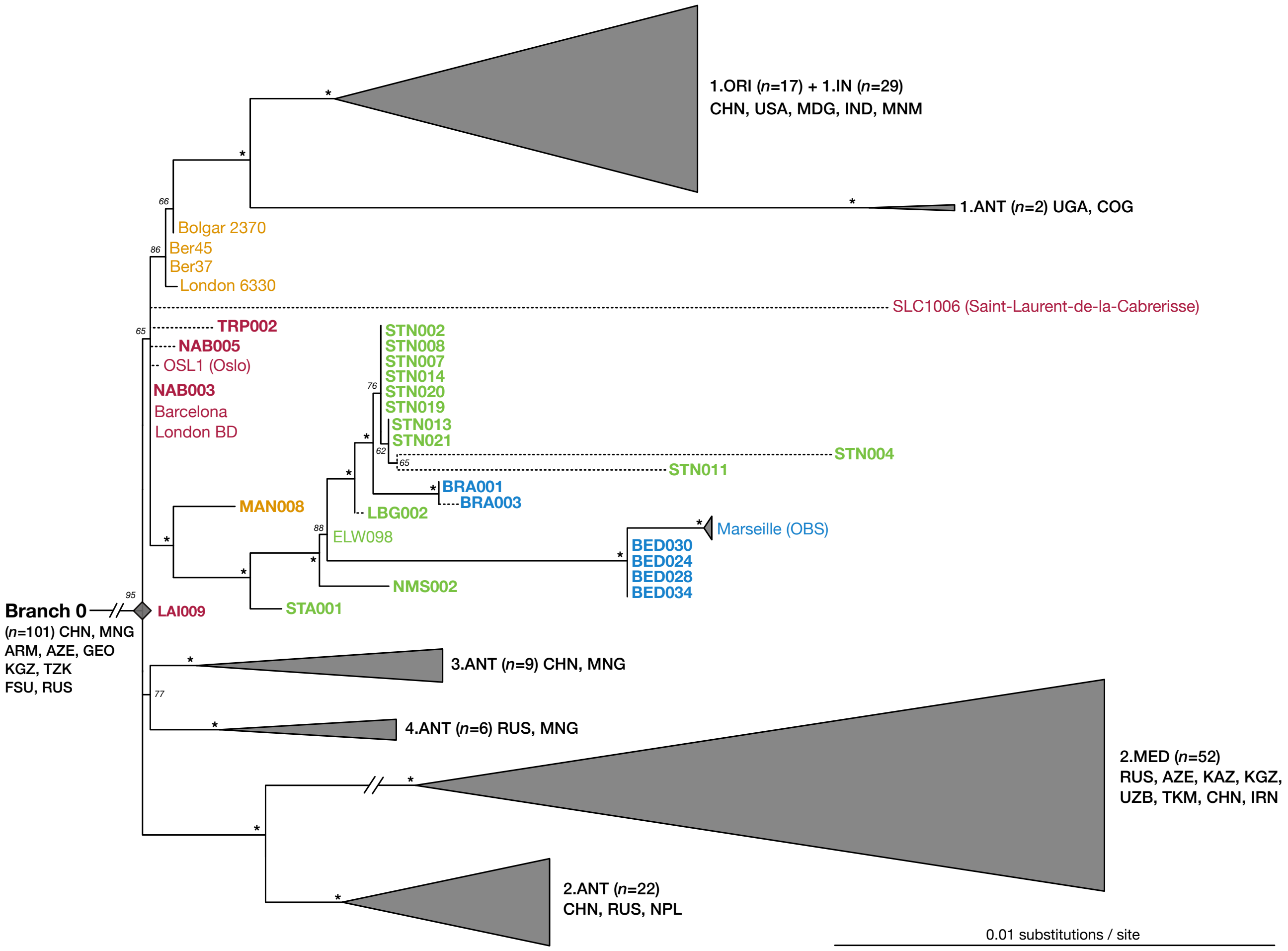

Supplementary figure 14 – A maximum likelihood phylogeny with 98% partial deletion was generated using RaxML[2]. The figure shows a graphical representation of Branches 1-4, and more specifically the phylogenetic positioning of all previously published and new second pandemic strains (14th-18th centuries). 5,736 SNP positions were used to generate the phylogeny. 1000 bootstrap iterations were carried out to determine node support. Sub-clades were collapsed to enhance tree clarity. Numbers in brackets indicate the number of strains contained in each collapsed branch. Isolates that showed evidence of false-positive SNPs to be influencing their branch lengths have their branches represented as dotted lines. Geographic abbreviations of modern strain isolation locations are as follows: China (CHN), United States of America (USA), Madagascar (MDG), India (IND), Myanmar (MNM), Congo (COG), Uganda (UGA), Mongolia (MNG), Nepal (NPL), Iran (IRN), Kazakhstan (KAZ), Kyrgyzstan (KGZ), Armenia (ARM), Georgia (GEO), Azerbaijan (AZE), Uzbekistan (UZB), Turkmenistan (TKM), Russia (RUS), as well as unspecified regions of the Former Soviet Union (FSU).

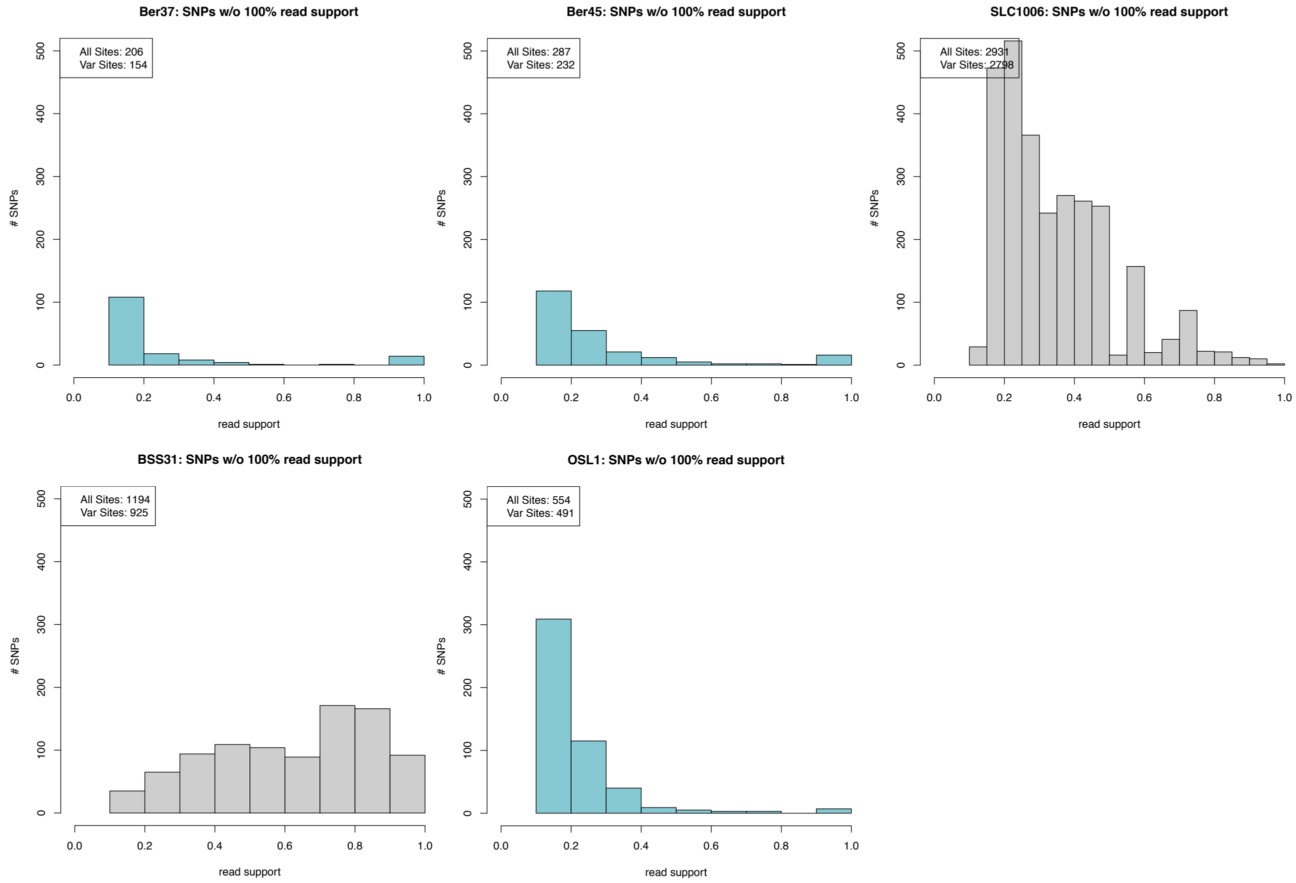

Supplementary figure 15 - Histograms showing the distribution of SNP allele frequencies in the 14th-century *Y. pestis* genomes from Namouchi et al[16]. Histograms shown in grey represent genomes that were excluded from further analysis due to high heterozygosity. The histograms were constructed using R version 3.4.1[15].

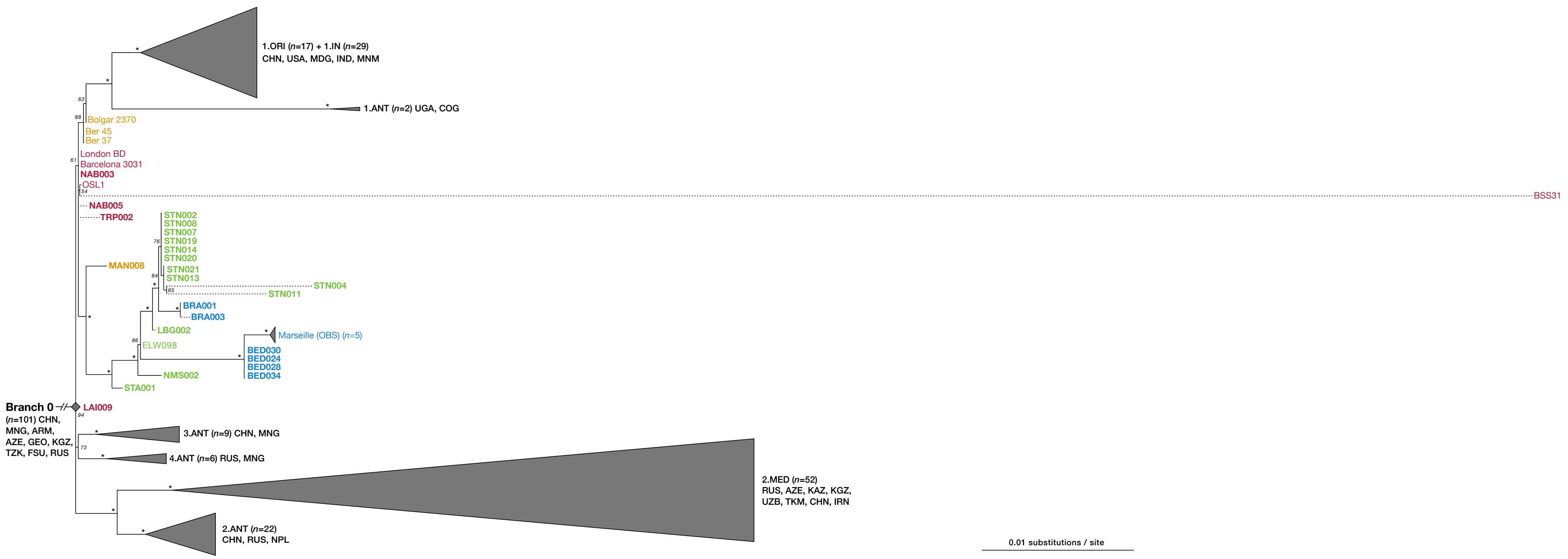

Supplementary figure 16 – A maximum likelihood phylogeny with 98% partial deletion was generated using RaxML[2] in order to determine the phylogenetic positioning of BSS31. Note that the low coverage of BSS31 (3-fold) appears to have affected the phylogenetic resolution and topologies in certain parts of the tree. For a more robust phylogeny (excluding BSS31), refer to Figure 2 in the main text. 5,867 SNP positions were used for generating the depicted tree. 1000 bootstrap iterations were carried out to determine node support. Sub-clades were collapsed to enhance tree clarity. Numbers in brackets indicate the number of strains comprising each collapsed branch. Isolates that showed evidence of environmental contamination to be influencing their SNP assignment have their branches represented by dotted lines. Geographic abbreviations of modern strain isolation locations are as follows: China (CHN), United States of America (USA), Madagascar (MDG), India (IND), Myanmar (MNM), Congo (COG), Uganda (UGA), Mongolia (MNG), Nepal (NPL), Iran (IRN), Kazakhstan (KAZ), Kyrgyzstan (KGZ), Armenia (ARM), Georgia (GEO), Azerbaijan (AZE), Uzbekistan (UZB), Turkmenistan (TKM), Russia (RUS), as well as unspecified regions of the Former Soviet Union (FSU).

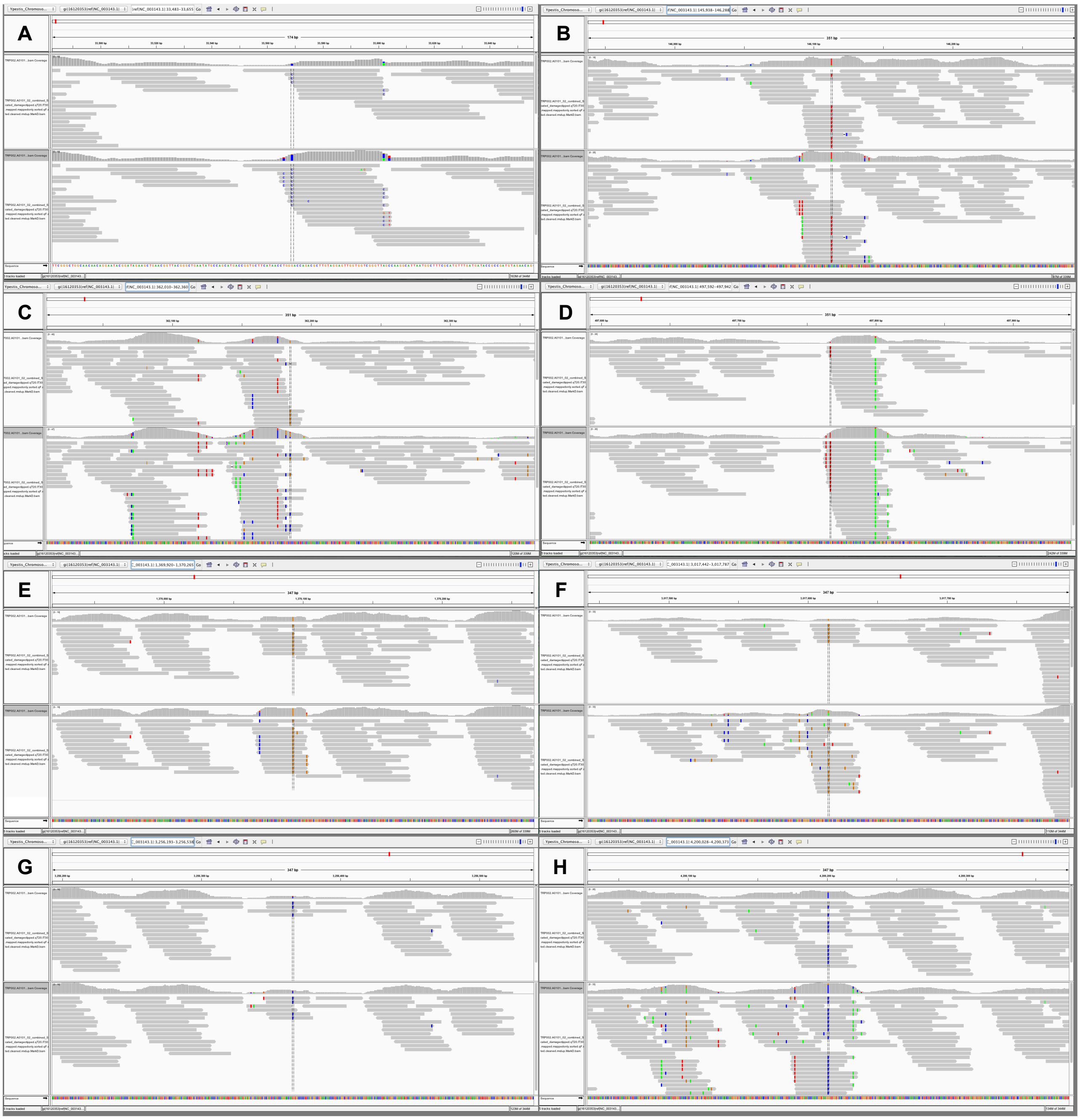

Supplementary figure 17 – Visual inspection of the eight private SNP positions identified in TRP002. The screenshots are made in IGV[17], with the cursor placed on the genomic position where each SNP was identified. BWA mapping was carried out using both stringent (A-H upper panels, -n 0.1) and lenient parameters (A-H lower panels, -n 0.01).

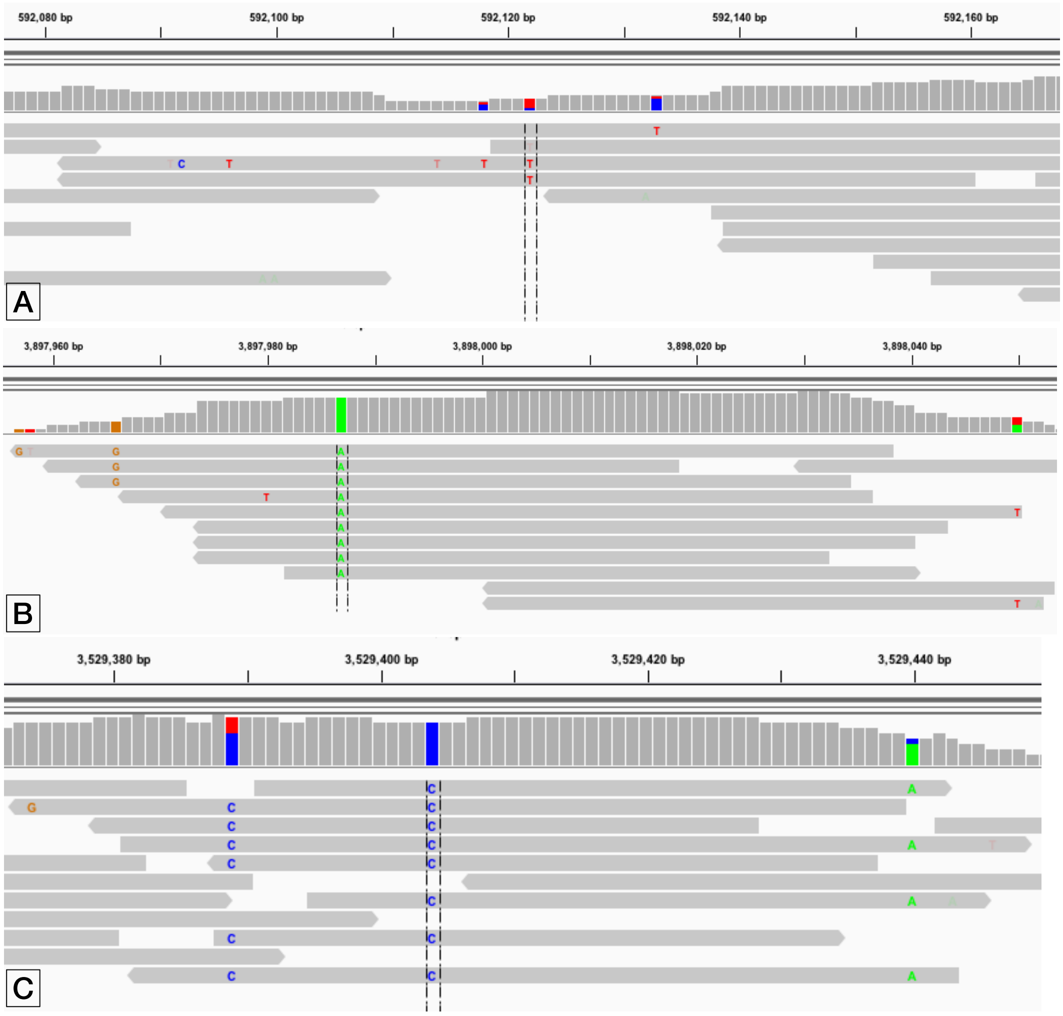

Supplementary figure 18 – Visual inspection of private SNPs in the previously published Black Death-associated *Y. pestis* genomes OSL1 and BSS31[16]. SNPs were inspected using the program IGV[17] (screenshots shown). Panel (A): C592122T indicates a private SNP identified in this study during re-analysis of OSL1, whereas (B): T3897987A and (C): T3529404C indicate previously identified private SNPs in BSS31[16].

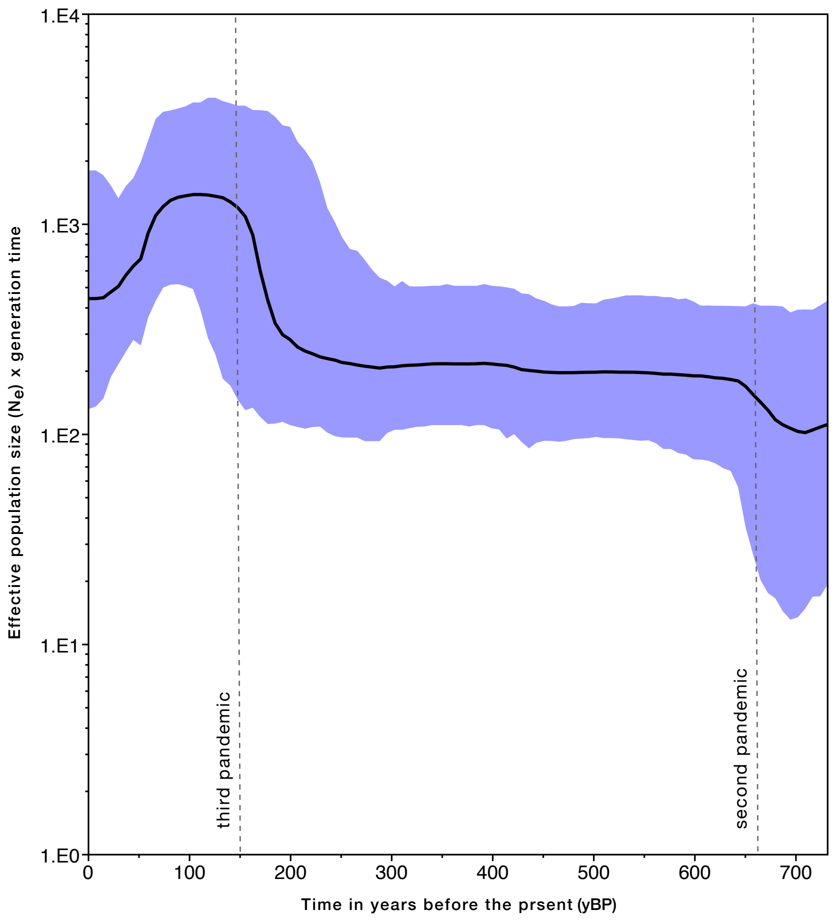

Supplementary figure 19 – Bayesian coalescent skyline. The figure is a depiction of effective population size *(N_e)* changes across the history of *Y. pestis* Branch 1 (*n*=80), as estimated by the coalescent skyline model[18] implemented in BEAST v1.8[19]. The plot was produced in Tracer v1.6 (http://tree.bio.ed.ac.uk/software/tracer/), after subsampling the MCMC states at lower frequency (subsampling every 100,000 states) using LogCombiner[19]. Dotted lines represent the proposed initiations of the second (1346 AD) and third (1855 AD) plague pandemics. The divergence date for Branch 1 was estimated to 731 yBP (95% HPD: 672 - 823).

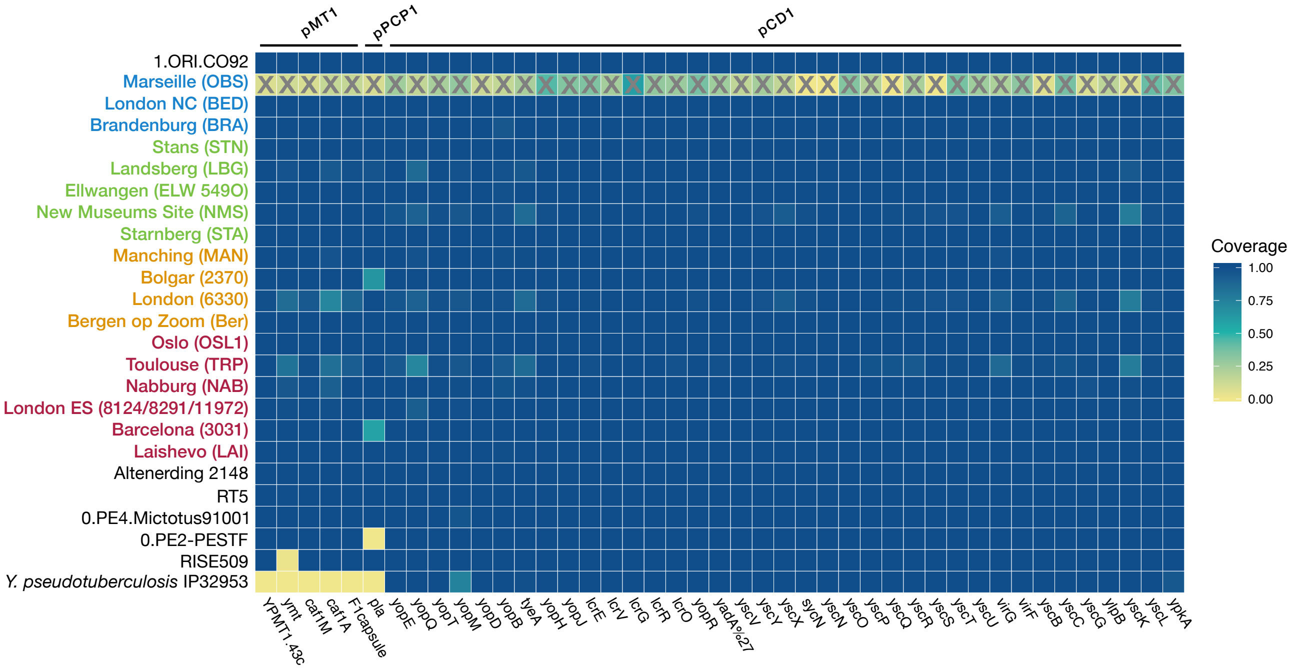

Supplementary figure 20 – Analysis of coverage across virulence-associated and evolutionary determinant genes located on the pCD1, pMT1 and pPCP1 plasmids of *Y. pestis*. The presence/absence of previously identified genes[20,21] was assessed for the newly reconstructed second pandemic genomes in relation to previously published representatives of ancient and modern strains. The heatmap was constructed using the ggplot2[22] package in R version 3.4.1[15]. Genomes are arranged from top to bottom according to their inferred divergence ages (from youngest to oldest), and all second pandemic strains are shown in red, orange, green or blue based on their age (see Figure 1). A lack of coverage across the plasmids in the Marseille OBS isolates is apparent since those regions were not used as part of the probe set for enrichment of these loci and are marked here with "X"[23]. Instead, missing regions in all other isolates represent real gene absences.

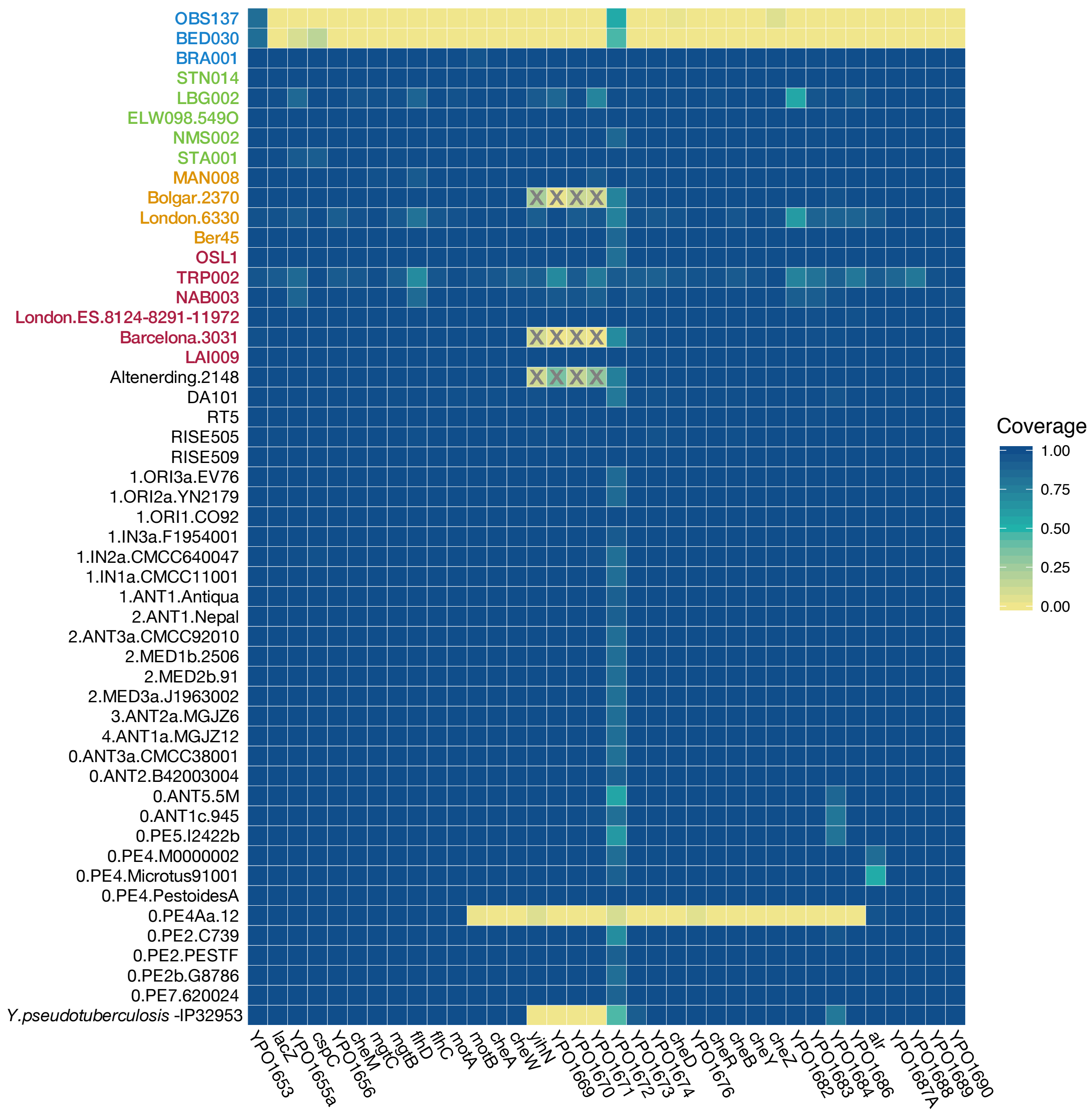

Supplementary figure 21 – Analysis of coverage across genes identified as absent in 17th-18th century second pandemic genomes BED and OBS. The presence or absence of genes located on the *Y. pestis* chromosome in representative ancient and modern genomes is shown. Specifically, second pandemic isolates from all studied archaeological sites (one representative chosen when more than one identical genome was recovered from a site), one published first pandemic genome from Germany (Altenerding 2148), one 2nd- to 3rd-century genome from the Tian Shan region (DA101) and three Bronze Age genomes from Russia (RT5, RISE505, RISE509) are shown for comparison. In addition, modern representatives from all phylogenetic clades are shown. The heatmap was constructed using the ggplot2[22] package in R version 3.4.1[15]. Genomes are arranged from top to bottom according to their inferred divergence ages (from youngest to oldest), and all second pandemic strains are shown in red, orange, green or blue based on their age (see Figure 1). A lack of coverage across certain *Y. pestis*-specific regions in Altenerding 2148, Barcelona 3031, and Bolgar 2370 are marked here with "X" since they were not used as part of the probe set for their enrichment[10,24]. Instead, missing regions in all other isolates represent real gene absences.

Supplementary table 1 - Sample description and quantification of *pla* through qPCR

Sample Name	Site	Country	Archaeological IDs	Archaeological date	Cal 2-sigma (95.4%) radiocarbon date (cal AD)	*pla* qPCR quantification (copies/µl)
BED030.A0102	New Churchyard "Bedlam", London	United Kingdom	8127	1600-1700	1560–1635 (combined date)	62.60
BED028.A0102	New Churchyard "Bedlam", London	United Kingdom	8103	1600-1700	1560–1635 (combined date)	15.70
BED034.A0102	New Churchyard "Bedlam", London	United Kingdom	8198	1600-1700	1560–1635 (combined date)	5.90
BED024.A0102	New Churchyard "Bedlam", London	United Kingdom	8052	1600-1700	1560–1635 (combined date)	1.38
BED038.A0102	New Churchyard "Bedlam", London	United Kingdom	8216	1600-1700	1560–1635 (combined date)	0.65
BRA001.A0101	Domlinden 12, Brandenburg	Germany	1*	1618-1648	N/A	1.64
BRA003.A0101	Domlinden 12, Brandenburg	Germany	3*	1618-1648	N/A	1.02
LAI009.A0101	Laishevo III, Laishevo	Russia	RT88 (burial 27B)	1300-1400	N/A	140.20
LAI010.A0101	Laishevo III, Laishevo	Russia	RT89 (burial 10A)	1300-1400	N/A	1.14
LBG002.A0101	Kirchhof St. Johannis, Landsberg	Germany	Bef. 460	N/A	1455-1632	1.52
LBG005.A0101	Kirchhof St. Johannis, Landsberg	Germany	Bef. 572	N/A	N/A	0.02
LBG007.A0101	Kirchhof St. Johannis, Landsberg	Germany	Bef. 598	N/A	N/A	0.32
MAN008.B0101	St. Leonhardi, Manching-Pichl	Germany	MPS03-I*	N/A	1283-1390	171.80
MAN015.A0101	St. Leonhardi, Manching-Pichl	Germany	MP56-X*	N/A	N/A	58.58
NAB005.A/B0101	"Sankt Johans Freidhof" Nabburg	Germany	471	N/A	1298-1398	0.043/2.174
NAB003.A/B0101	"Sankt Johans Freidhof" Nabburg	Germany	452	N/A	1292-1392	19.5/17.18
NAB004.A/B0101	"Sankt Johans Freidhof" Nabburg	Germany	457	N/A	1317-1420	0.1069/0.1069
NAB002.A0101	"Sankt Johans Freidhof" Nabburg	Germany	451	N/A	N/A	0.5980
STA001.A0101	Possenhofener Str. 3, Starnberg	Germany	207	1433-1523	1420-1630	3.60
STN014.A0101	Nägeligasse, Stans	Switzerland	Grave 104 (mult. bur. 2)	N/A	1485-1635 (combined date)	129.60
STN020.A0101	Nägeligasse, Stans	Switzerland	Grave 124 (mult. bur. 2)	N/A	1485-1635 (combined date)	66.93
STN021.A0101	Nägeligasse, Stans	Switzerland	Grave 125 (mult. bur. 2)	N/A	1485-1635 (combined date)	11.71
STN019.A0101	Nägeligasse, Stans	Switzerland	Grave 123 (mult. bur. 2)	N/A	1485-1635 (combined date)	34.75
STN007.A0101	Nägeligasse, Stans	Switzerland	Grave 97/POS.251 (mult. bur. 1)	N/A	1485-1635 (combined date)	26.47
STN002.A0101	Nägeligasse, Stans	Switzerland	Grave 71 (mult. bur. 1)	N/A	1485-1635 (combined date)	10.65
STN008.A0101	Nägeligasse, Stans	Switzerland	Grave 98/POS.250 (mult. bur. 1)	N/A	1485-1635 (combined date)	15.87
STN013.A0101	Nägeligasse, Stans	Switzerland	Grave 85 (mult. bur. 1)	N/A	1485-1635 (combined date)	3.07
STN011.A0101	Nägeligasse, Stans	Switzerland	Grave 102 (mult. bur. 2)	N/A	1485-1635 (combined date)	2.07
STN004.A0101	Nägeligasse, Stans	Switzerland	Grave 91 (mult. bur. 1)	N/A	1485-1635 (combined date)	1.24
STN032.A0101	Nägeligasse, Stans	Switzerland	Grave 167 (mult. bur. 3)	N/A	1485-1635 (combined date)	1.89
STN031.A0101	Nägeligasse, Stans	Switzerland	Grave 165 (mult. bur. 3)	N/A	1485-1635 (combined date)	12.52
STN005.A0101	Nägeligasse, Stans	Switzerland	Grave 92 (mult. bur. 1)	N/A	1485-1635 (combined date)	0.89
STN018.A0101	Nägeligasse, Stans	Switzerland	Grave 121 (mult. bur. 2)	N/A	1485-1635 (combined date)	0.17
STN012.A0101	Nägeligasse, Stans	Switzerland	Grave 103 (mult. bur. 1)	N/A	1485-1635 (combined date)	0.16
STN015.A0101	Nägeligasse, Stans	Switzerland	Grave 105 (mult. bur. 2)	N/A	1485-1635 (combined date)	0.13
STN026.A0101	Nägeligasse, Stans	Switzerland	Grave 134 (mult. bur. 2)	N/A	1485-1635 (combined date)	0.28
STN016.A0101	Nägeligasse, Stans	Switzerland	Grave 106 (mult. bur. 2)	N/A	1485-1635 (combined date)	0.40
TRP002.A0101	Trente-Six Ponts str. 16, Toulouse	France	Ind. 1352	1347-1350	1288-1394	0.02

*Individuals from Manching-Pichl and Brandenburg and der Havel where investigated in previous aDNA studies, where *Y. pestis* was detected by PCR[25-28]

Supplementary table 2 – Summarised HOPS statistics for the analysed samples from the Augustinian Friary in Cambridge (NMS).

**Sample ID**	**Archaeological ID**	**Total assigned reads in MALT**	**Reads assigned to the *Y. pseudotuberculosis* complex**	**Percentage C-to-T 5' end**	**Percentage G-to-A 3' end**	**Average fragment length**
NMS001	F. 190	1,574,633	110	3%	13%	49
NMS002	F. 230	2,572,436	3,795	15.1%	13.5%	49
NMS003	F. 355	589,608	434	20.3%	12.1%	51
NMS005	F. 310	860,459	50	0%	5.3%	50

Supplementary table 3 – Sequencing statistics after whole-genome *Y. pestis* capture for all samples that yielded coverage ≥ 1-fold

Sample IDs	Archaeological IDs	Pre-processed reads	Mapping reads	Unique mapping reads after quality filtering	Endogenous DNA (%)	Cluster factor	Mean coverage	Std. dev. coverage	Coverage ≥ 5X (%)	Average frag. length	Median frag. length	Percentage GC (%)
BED030.A0102	8127	19,242,747	7,335,361	3,624,482	36.2	1.9	80.1	35.1	93.6	102.9	101.0	48.5
BED028.A0102	8103	27,963,243	6,830,578	2,665,238	22.2	2.3	37.2	23.7	91.4	65.0	66.0	49.0
BED034.A0102	8198	29,358,235	3,489,557	1,371,698	10.5	2.2	18.3	10.3	89.1	62.2	62.0	49.2
BED024.A0102	8052	11,064,780	2,209,748	1,000,524	18.1	2.0	12.6	7.7	84.7	58.5	56.0	49.1
BED038.A0102	8216	31,240,177	1,974,588	458,397	5.3	3.6	4.9	4.5	44.3	50.0	49.0	47.6
BRA001.A0101	1*	20,535,183	5,297,674	2,387,557	23.2	2.0	23.8	12.7	92.0	46.4	44.0	47.5
BRA003.A0101	3*	43,129,525	2,568,078	849,170	3.4	1.7	9.1	5.5	79.2	49.8	46.0	47.4
LAI009.A0101	RT88 (burial 27B)	23,417,187	6,144,041	2,549,926	23.9	2.2	28.4	15.9	92.1	51.8	50.0	48.0
LAI010.A0101	RT89 (burial 10A)	64,934,571	1,269,665	133,256	1.0	4.8	1.3	1.4	2.8	43.9	42.0	48.4
LBG002.A0101	Bef. 460	5,696,055	1,793,631	621,713	27.9	2.6	7.2	5.3	66.4	54.2	53.0	49.9
MAN008.B0101	MPS03-I*	8,084,687	3,936,101	1,974,399	44.9	1.8	25.8	16.1	88.7	60.8	62.0	50.8
MAN015.A0101	MP56-X*	1,299,105	178,023	121,546	10.7	1.1	1.6	1.7	6.7	62.0	64.0	49.9
NAB005.A/B0101	471	66,111,306	2,757,864	786,575	2.9	2.4	8.3	5.6	73.3	49.1	47.0	47.5
NAB003.B0101	452	6,034,650	2,230,536	684,029	33.5	3.0	8.1	5.7	70.2	54.8	53.0	49.7
NAB004.A0101	457	79,055,317	1,491,516	202,056	0.7	2.8	1.9	1.8	8.2	42.8	41.0	46.6
NMS002.A0101	F. 230 / 507	68,353,310	2,022,552	855,185	2.3	1.8	12.5	5.5	91.7	67.9	72	47.3
NMS003.A0101	F. 355 / 517	22,641,646	2,550,272	289,755	9.8	7.7	4.2	2.6	42.6	67.7	66	47.1
STA001.A0101	207	28,712,935	3,283,300	1,110,049	9.9	2.6	11.7	7.2	84.3	49.0	46.0	44.7
STN014.A0101	Grave 104 (mult. bur.2)	15,736,877	8,069,865	3,822,030	48.2	2.0	55.3	26.6	93.0	67.3	74.0	48.9
STN020.A0101	Grave 124 (mult. bur.2)	7,368,937	3,502,272	2,020,769	44.3	1.6	28.2	16.9	90.3	64.8	70.0	48.5
STN021.A0101	Grave 125 (mult. bur.2)	8,852,058	3,441,583	1,588,442	35.1	2.0	21.7	13.8	88.6	63.7	66.0	48.5
STN019.A0101	Grave 123 (mult. bur.2)	5,534,501	2,114,414	1,325,076	35.3	1.5	18.7	11.8	87.1	65.8	72.0	49.1
STN007.A0101	Grave 97/POS.251 (mult. bur.1)	6,824,093	2,538,685	1,293,507	32.8	1.7	18.0	11.5	86.7	64.8	70.0	49.4
STN002.A0101	Grave 71 (mult. bur.1)	6,912,906	2,089,426	935,795	27.6	2.0	12.7	8.1	83.3	63.0	66.0	48.4
STN008.A0101	Grave 98/POS.250 (mult bur.1)	4,372,116	1,469,871	875,153	30.2	1.5	11.7	8.6	77.7	62.5	65.0	50.1
STN013.A0101	Grave 85 (mult. bur.2)	5,801,670	1,582,429	714,482	24.5	2.0	9.2	6.6	73.8	59.9	60.0	49.0
STN011.A0101	Grave 102 (mult. bur.1)	9,187,148	2,938,862	671,509	24.4	3.3	8.1	6.6	69.1	56.2	54.0	48.3
STN004.A0101	Grave 91 (mult. bur.1)	7,045,866	1,913,895	531,605	22.6	3.0	6.2	5.5	57.2	54.6	53.0	49.5
STN032.A0101	Grave 167 (mult. bur.3)	4,130,111	566,869	237,195	9.0	1.6	3.4	2.9	29.5	65.8	73.0	49.3
STN031.A0101	Grave 165 (mult. bur.3)	3,217,122	337,111	127,950	6.7	1.7	1.8	1.8	8.1	64.3	68.0	49.7
STN005.A0101	Grave 92 (mult. bur.1)	3,678,388	299,437	108,298	6.0	2.0	1.3	1.5	3.7	55.4	54.0	48.5
STN018.A0101	Grave 121 (mult. bur.2)	5,528,516	326,360	94,481	4.0	2.3	1.2	1.5	3.5	58.5	58.0	48.4
STN012.A0101	Grave 103 (mult. bur.1)	5,680,142	432,404	86,278	4.9	3.2	1.1	1.4	2.6	61.3	63.0	48.1
TRP002.A0101/02	Ind. 1352	22,619,908	5,173,193	632,303	19.8	7.1	5.9	5.4	50.9	43.2	42.0	48.7
ELW098.A0101	549_O**	48,518,869	5,078,186	1,435,225	9.0	3.0	14.2	7.8	89.1	46.0	44.0	46.6

* Samples from sites that were analysed in previous aDNA studies, where *Y. pestis* was detected by PCR[25-28]
**Previously published Ellwangen individual[24]

Supplementary table 4 – Genes affected by the 49 kb deletion identified among BED and OBS isolates (coordinates on CO92 reference genome: 1,879,467 – 1,928,869)

Gene name	Start position (CO92)	End position (CO92)	Function
YPO1690	1928171	1928404	hypothetical protein
YPO1689	1926760	1928016	lipoprotein
YPO1688	1926119	1926367	hypothetical protein
YPO1687A	1925346	1925540	hypothetical protein
alr	1923715	1924944	alanine racemase: converts L-alanine to D-alanine which is used in cell wall biosynthesis
YPO1686	1922143	1923096	hypothetical protein
YPO1684	1919412	1921622	surface protein
YPO1683	1918291	1919055	N-acetylmuramoyl-L-alanine amidase
YPO1682	1918040	1918159	hypothetical protein
cheZ	1917082	1917726	chemotaxis regulator
cheY	1916683	1917072	chemotaxis regulatory protein
cheB	1915533	1916582	chemotaxis-specific methylesterase
cheR	1914661	1915533	chemotaxis methyltransferase
YPO1676	1912878	1914488	pseudogene
cheD	1911021	1912694	methyl-accepting chemotaxis protein
YPO1674	1910079	1910780	hypothetical protein
YPO1673	1907134	1910010	hypothetical protein
YPO1672	1902800	1906606	hypothetical protein
YPO1671	1901648	1902325	DNA-binding protein
YPO1670	1900484	1901470	hypothetical protein
YPO1669	1900110	1900487	hypothetical protein
yihN	1898862	1900097	hypothetical protein
cheW	1898073	1898570	purine-binding chemotaxis protein
cheA	1895739	1897910	chemotaxis protein
motB	1894445	1895728	flagellar motor protein
motA	1893561	1894448	flagellar motor protein
flhC	1892772	1893356	transcriptional activator
flhD	1892411	1892771	pseudogene
mgtB	1888533	1891232	Mg^{2+} transport ATPase protein B
mgtC	1887422	1888132	Mg^{2+} transport ATPase protein C
cheM	1884574	1886275	pseudogene
YPO1656	1884200	1884391	hypothetical protein
cspC	1883773	1883985	cold shock protein
YPO1655a	1883592	1884034	ncRNA-cspA thermoregulator
lacZ	1880050	1883232	beta-D-galactosidase
YPO1653	1878633	1879643	zinc-binding dehydrogenase

Supplementary References

1 Huebler, R. *et al.* HOPS: Automated detection and authentication of pathogen DNA in archaeological remains. *bioRxiv*, 534198 (2019).

2 Stamatakis, A. RAxML version 8: a tool for phylogenetic analysis and post-analysis of large phylogenies. *Bioinformatics* **30**, 1312-1313, doi:10.1093/bioinformatics/btu033 (2014).

3 Kutyrev, V. V. *et al.* Phylogeny and Classification of Yersinia pestis through the Lens of Strains from the Plague Foci of Commonwealth of Independent States. *Front Microbiol* **9** (2018).

4 Eroshenko, G. A. *et al.* Yersinia pestis strains of ancient phylogenetic branch 0. ANT are widely spread in the high-mountain plague foci of Kyrgyzstan. *PloS one* **12**, e0187230 (2017).

5 Zhgenti, E. *et al.* Genome Assemblies for 11 Yersinia pestis Strains Isolated in the Caucasus Region. *Genome Announc* **3**, e01030-01015, doi:10.1128/genomeA.01030-15 (2015).

6 Kislichkina, A. A. *et al.* Six Whole-Genome Assemblies of Yersinia pestis subsp. microtus bv. ulegeica (Phylogroup 0. PE5) Strains Isolated from Mongolian Natural Plague Foci. *Genome announcements* **6**, e00536-00518 (2018).

7 Kislichkina, A. A. *et al.* Nine Whole-Genome Assemblies of Yersinia pestis subsp. microtus bv. Altaica Strains Isolated from the Altai Mountain Natural Plague Focus (No. 36) in Russia. *Genome announcements* **6**, e01440-01417 (2018).

8 Kislichkina, A. A. *et al.* Nineteen Whole-Genome Assemblies of Yersinia pestis subsp. microtus, Including Representatives of Biovars caucasica, talassica, hissarica, altaica, xilingolensis, and ulegeica. *Genome Announc* **3**, e01342-01315, doi:10.1128/genomeA.01342-15 (2015).

9 Cui, Y. *et al.* Historical variations in mutation rate in an epidemic pathogen, Yersinia pestis. *Proceedings of the National Academy of Sciences* **110**, 577-582 (2013).

10 Feldman, M. *et al.* A high-coverage Yersinia pestis genome from a sixth-century Justinianic plague victim. *Mol. Biol. Evol.* **33**, 2911-2923 (2016).

11 de Barros Damgaard, P. *et al.* 137 ancient human genomes from across the Eurasian steppes. *Nature* **557**, 369 (2018).

12 Rasmussen, S. *et al.* Early Divergent Strains of Yersinia pestis in Eurasia 5,000 Years Ago. *Cell* **163**, 571-582, doi:10.1016/j.cell.2015.10.009 (2015).

13 Spyrou, M. A. *et al.* Analysis of 3800-year-old Yersinia pestis genomes suggests Bronze Age origin for bubonic plague. *Nat. Commun.* **9**, 2234 (2018).

14 Chain, P. S. *et al.* Insights into the evolution of Yersinia pestis through whole-genome comparison with Yersinia pseudotuberculosis. *Proc Natl Acad Sci U S A* **101**, 13826-13831, doi:10.1073/pnas.0404012101 (2004).

15 R Core Team. R: A language and environment for statistical computing. *R Foundation for Statistical Computing, Vienna, Austria* (2015).

16 Namouchi, A. *et al.* Integrative approach using Yersinia pestis genomes to revisit the historical landscape of plague during the Medieval Period. *Proceedings of the National Academy of Sciences*, 201812865 (2018).

17 Thorvaldsdóttir, H., Robinson, J. T. & Mesirov, J. P. Integrative Genomics Viewer (IGV): high-performance genomics data visualization and exploration. *Brief. Bioinformatics* **14**, 178-192 (2013).

18 Drummond, A. J., Rambaut, A., Shapiro, B. & Pybus, O. G. Bayesian coalescent inference of past population dynamics from molecular sequences. *Mol. Biol. Evol.* **22**, 1185-1192, doi:10.1093/molbev/msi103 (2005).

19 Drummond, A. J. & Rambaut, A. BEAST: Bayesian evolutionary analysis by sampling trees. *BMC Evol. Biol.* **7**, 214 (2007).

20 Zhou, D. & Yang, R. Molecular Darwinian evolution of virulence in Yersinia pestis. *Infect. Immun.* **77**, 2242-2250, doi:10.1128/IAI.01477-08 (2009).

21 Zhou, D. *et al.* Genetics of metabolic variations between Yersinia pestis biovars and the proposal of a new biovar, microtus. *J. Bacteriol.* **186**, 5147-5152 (2004).

22 Wickham, H. *ggplot2: elegant graphics for data analysis*. (Springer, 2016).

23 Bos, K. I. *et al.* Eighteenth century Yersinia pestis genomes reveal the long-term persistence of an historical plague focus. *Elife* **5**, e12994, doi:10.7554/eLife.12994 (2016).
24 Spyrou, M. A. *et al.* Historical Y. pestis genomes reveal the European Black Death as the source of ancient and modern plague pandemics. *Cell host & microbe* **19**, 874-881 (2016).
25 Wiechmann, I., Harbeck, M. & Grupe, G. Yersinia pestis DNA sequences in late medieval skeletal finds, Bavaria. *Emerging Infect. Dis.* **16**, 1806 (2010).
26 Seifert, L. *et al.* Genotyping Yersinia pestis in Historical Plague: Evidence for Long-Term Persistence of Y. pestis in Europe from the 14th to the 17th Century. *PLoS One* **11**, e0145194, doi:10.1371/journal.pone.0145194 (2016).
27 Seifert, L. *et al.* Strategy for sensitive and specific detection of Yersinia pestis in skeletons of the Black Death pandemic. *Plos one* **8**, e75742 (2013).
28 Garrelt, C. & Wiechmann, I. Detection of Yersinia pestis DNA in early and late medieval Bavarian burials. *Decyphering ancient bones; the research potential of bioarchaeological collections. Documenta Archaeobiologiae*, 247-254 (2003).
29 Paya, D. & Catalo, J. *Le cimetière Saint-Michel de Toulouse*. (CNRS, 2011).
30 Hartle, R., Carty, N., Henderson, M., Knox E. L., Walker, D. The New Churchyard: from Moorfields marsh to Bethlem burial ground, Brokers Row and Liverpool Street. *Crossrail Archaeol Ser* **10** (2017).
31 Keily, J. *Tunnel: the archaeology of Crossrail*. (2017).
32 Cressford, C. Former Old Examination Hall, North Range Buildings, New Museums Site, Cambridge: an archaeological excavation *Cambridge Archaeological Unit report no. 1377* (2017).
33 Hensch, M. *Sankt Johans Freidhof in Nabburg - Gewöhnliche und ungewöhnliche Einblicke in die spätmittelalterliche Begräbniskultur Ostbayerns.*, 423–440 (2014).
34 Garrelt, C. *Molekulargenetische Untersuchung der Bestatteten eines vermuteten Pestfriedhofs des 14. Jahrhunderts (Manching-Pichl)*, Diplomarbeit LMU München, (2002).
35 Later, C. Merowingerzeitliche Tuffplattengräber und frühmittelalterliche Kirchenbauten – Zu den Anfängen der ehemaligen Pfarrkirche St. Benedikt in Starnberg. *Bericht Der Bayerischen Bodendenkmalpflege* **52**, 373–402 (2010).
36 Lichtenstern, A. Landberg am Lech. *Geschichte und Kultur, Mering* (2012).
37 Schreiber, J., Carlichi-Witjes, N., von Heyking, K. & Immler, F. 1507-1806 : Hunderte Gräber vom Kirchhof St. Johannis in Landsberg am Lech. *Das Archäologische Jahr in Bayern*, 181–182 (2016).
38 Schreiber, J. Grabungen im Neuzeitlichen Kirchhof St. Johannis an der Brudergasse. *Dokumenta historiae* **18**, 105-145 (2017).
39 Krämer, D. Bevölkerung und Wegenetz: Leben in Abgeschiedenheit. *Geschichte des Kantons Nidwalden. Von der Urzeit bis 1850. Band 1.*, 160-172 (2014).
40 Homberger, V. R., K. Stans NW, Kreuzung Nägeligasse /Knirigasse. *Jahrbuch Archäologie Schweiz* **100**, 276-277 (2017).
41 Dalitz, S., Grupe, G. & Jungklaus, B. Das kleinste Massengrab Brandenburgs. Drei Tote aus dem Dreißigjährigen Krieg auf der Dominsel der Stadt Brandenburg an der Havel. *Historischer Verein Brandenburg (Havel) eV* **21**, 2011-2012 (2012).
