# Supplementary material for: Phylogeography of the second plague pandemic revealed through analysis of historical Yersinia pestis genomes
Source: Nat Commun. 2019 Oct 2;10:4470. doi: 10.1038/s41467-019-12154-0 (PMC6775055; doi:10.1038/s41467-019-12154-0)
Supplement: Supplementary file 3 — Description of Additional Supplementary Files [file 41467_2019_12154_MOESM3_ESM.docx]

**Description of Additional Supplementary Files**

File Name: **Supplementary Data 1**

Description: Variant genomic positions identified across previously published and new second pandemic *Y. pestis* genomes sequenced to date

File Name: **Supplementary Data 2**

Description: Assessment of unique SNPs in all newly reconstructed genomes that exhibited a minimum of 50% genomic coverage at 5-fold. Genomes that are not listed within the table did not yield any private SNP calls (LAI009, STN014, STN021, STN019, STN007, STN002, STN008, STN013, BED034, BED024).

File Name: **Supplementary Data 3**

Description: SNP profile of the low-coverage NMS003 isolate

File Name: **Supplementary Data 4**

Description: BLASTn analysis of reads overlapping private SNP position T3897987A, previously identified in the BSS31 genome

File Name: **Supplementary Data 5**

Description: BLASTn analysis of reads overlapping private SNP position T3529404C, previously identified in the BSS31 genome

File Name: **Supplementary Data 6**

Description: Isolation, radiocarbon and archaeological dates used for BEAST v1.8 substitution rate variation analysis

File Name: **Supplementary Data 7**

Description: Gene annotation and effects of SNPs located on the branch between the Ellwangen (ELLW) and New Churchyard (BED) strains

File Name: **Supplementary Data 8**

Description: Gene annotations and effects of variants unique to 1.ANT strains

File Name: **Supplementary Data 9**

Description: Gene annotations and effects of SNPs located on the branch between the 1.IN and 1.ORI strains
