# Supplementary material for: Phylogeography of the second plague pandemic revealed through analysis of historical Yersinia pestis genomes
Source: Nat Commun. 2019 Oct 2;10:4470. doi: 10.1038/s41467-019-12154-0 (PMC6775055; doi:10.1038/s41467-019-12154-0)
Supplement: Supplementary file 13 — Reporting Summary [file 41467_2019_12154_MOESM13_ESM.pdf]

nature research

Corresponding author(s): Maria A. Spyrou
Kirsten I. Bos
Johannes Krause

Last updated by author(s): Jul 29, 2019

Reporting Summary

Nature Research wishes to improve the reproducibility of the work that we publish. This form provides structure for consistency and transparency in reporting. For further information on Nature Research policies, see Authors & Referees and the Editorial Policy Checklist.

Statistics

For all statistical analyses, confirm that the following items are present in the figure legend, table legend, main text, or Methods section.

n/a	Confirmed	
☐	☒	The exact sample size (*n*) for each experimental group/condition, given as a discrete number and unit of measurement
☒	☐	A statement on whether measurements were taken from distinct samples or whether the same sample was measured repeatedly
☒	☐	The statistical test(s) used AND whether they are one- or two-sided<br>*Only common tests should be described solely by name; describe more complex techniques in the Methods section.*
☒	☐	A description of all covariates tested
☒	☐	A description of any assumptions or corrections, such as tests of normality and adjustment for multiple comparisons
☐	☒	A full description of the statistical parameters including central tendency (e.g. means) or other basic estimates (e.g. regression coefficient) AND variation (e.g. standard deviation) or associated estimates of uncertainty (e.g. confidence intervals)
☒	☐	For null hypothesis testing, the test statistic (e.g. *F*, *t*, *r*) with confidence intervals, effect sizes, degrees of freedom and *P* value noted<br>*Give P values as exact values whenever suitable.*
☐	☒	For Bayesian analysis, information on the choice of priors and Markov chain Monte Carlo settings
☒	☐	For hierarchical and complex designs, identification of the appropriate level for tests and full reporting of outcomes
☐	☒	Estimates of effect sizes (e.g. Cohen's *d*, Pearson's *r*), indicating how they were calculated

Our web collection on statistics for biologists contains articles on many of the points above.

Software and code

Policy information about availability of computer code

Data collection	No specialized code was used for data retrieval. All comparative data used for this study was collected from published work (publications listed within Methods section) and was downloaded from public databases such as the National Center for Biotechnology Information (NCBI), and the European Nucleotide Archive (ENA).
Data analysis	The software used in this study is the following:<br>EAGER v1.92.55 (Peltzer et al., 2016); AdapterRemoval v2 (Schubert et al., 2016); BWA v0.7.12 (Li et al., 2010); BWA-MEM (Li et al., 2013) Integrative Genomics Viewer (IGV v2.3.57) (Thorvaldsdóttir et al., 2013); MarkDuplicates (http://broadinstitute.github.io/picard/); SAMtools (http://samtools.sourceforge.net/); bedtools (Quinlan et al., 2010); GATK v3.5 (DePristo et al., 2011); MultivcfAnalyzer v0.85 (https://github.com/alexherbig/MultiVCFAnalyzer, Bos et al., 2014); MALT v040 (Vagene et al., 2018); HOPS (Huelbler et al, 2019); SNPEvaluation (https://github.com/andreasKroepelin/SNP_Evaluation); snpEff v3.1i (Cingolani et al., 2012); BEAST v1.8 (Drummond et al., 2007); Tracer v1.6 (http://tree.bio.ed.ac.uk/software/tracer/); LogCombiner v1.8.1 (Drummond et al., 2007); TreeAnnotator v1.8.1 (Drummond et al., 2007); FigTree v1.4.2 (http://tree.bio.ed.ac.uk/software/figtree/); RaxML v8.2.9 (Stamatakis et al, 2014); MEGA7 (Kumar et al., 2016); TempEst v1.5.1 (http://tree.bio.ed.ac.uk/software/tempest/); R version 3.4.1 (R Core Team, 2015); ggplot2 (Wickham, 2016)<br><br>All software used to analyze and visualize the presented dataset is publicly available and is described within the Methods section.

For manuscripts utilizing custom algorithms or software that are central to the research but not yet described in published literature, software must be made available to editors/reviewers. We strongly encourage code deposition in a community repository (e.g. GitHub). See the Nature Research guidelines for submitting code & software for further information.

Data

Policy information about availability of data

All manuscripts must include a data availability statement. This statement should provide the following information, where applicable:

- Accession codes, unique identifiers, or web links for publicly available datasets
- A list of figures that have associated raw data
- A description of any restrictions on data availability

Raw sequencing reads produced for this study are available at the European Nucleotide Archive (ENA) under accession nr: PRJEB29990.

Field-specific reporting

Please select the one below that is the best fit for your research. If you are not sure, read the appropriate sections before making your selection.

☒ Life sciences ☐ Behavioural & social sciences ☐ Ecological, evolutionary & environmental sciences

For a reference copy of the document with all sections, see nature.com/documents/nr-reporting-summary-flat.pdf

Life sciences study design

All studies must disclose on these points even when the disclosure is negative.

Sample size	Our sample size was determined by the number of human remains excavated from each of the studied archaeological sites, as well as by the number of specimens that we were permitted access to by the corresponding archaeological units. In addition, the number of genomes generated for this study was determined by the amount of specimens showing traces of ancient Y. pestis DNA.
Data exclusions	Low coverage or low quality genomes were excluded from parts of the analysis. Further details can be found on the Methods and Supplementary Information sections.
Replication	Initial qPCR screening results were subsequently verified by whole-genome production from the same DNA extracts, with all presented ancient genomes having a 1-fold to 80-fold average genomic coverage. In addition, evolutionary inferences were assessed through multiple iteration testing, as well as through the use of different phylogenetic methods (see Methods section for further details).
Randomization	No randomization testing was applied.
Blinding	Blinding was not applied in this study as no human or animal participants were used.

Reporting for specific materials, systems and methods

We require information from authors about some types of materials, experimental systems and methods used in many studies. Here, indicate whether each material, system or method listed is relevant to your study. If you are not sure if a list item applies to your research, read the appropriate section before selecting a response.

Materials & experimental systems

n/a	Involved in the study	
☒	☐	Antibodies
☒	☐	Eukaryotic cell lines
☒	☐	Palaeontology
☒	☐	Animals and other organisms
☒	☐	Human research participants
☒	☐	Clinical data

Methods

n/a	Involved in the study	
☒	☐	ChIP-seq
☒	☐	Flow cytometry
☒	☐	MRI-based neuroimaging
